# Cost-effectiveness of Mechanical Thrombectomy More Than 6 Hours After Symptom Onset Among Patients With Acute Ischemic Stroke

**DOI:** 10.1001/jamanetworkopen.2020.12476

**Published:** 2020-08-25

**Authors:** Anne-Claire Peultier, Ankur Pandya, Richa Sharma, Johan L. Severens, W. Ken Redekop

**Affiliations:** 1 Erasmus School of Health Policy and Management, Erasmus University Rotterdam, Rotterdam, the Netherlands; 2 Department of Health Policy and Management, Harvard T.H. Chan School of Public Health, Boston, Massachusetts; 3 Department of Neurology, Yale School of Medicine, New Haven, Connecticut; 4 Institute for Medical Technology Assessment, Erasmus University Rotterdam, Rotterdam, the Netherlands

## Abstract

**Importance:**

Two 2018 randomized controlled trials (DAWN and DEFUSE 3) demonstrated the clinical benefit of mechanical thrombectomy (MT) more than 6 hours after onset in acute ischemic stroke (AIS). Health-economic evidence is needed to determine whether the short-term health benefits of late MT translate to a cost-effective option during a lifetime in the United States.

**Objective:**

To compare the cost-effectiveness of 2 strategies (MT added to standard medical care [SMC] vs SMC alone) for various subgroups of patients with AIS receiving care more than 6 hours after symptom onset.

**Design, Setting, and Participants:**

This economic evaluation study used the results of the DAWN and DEFUSE 3 trials to populate a cost-effectiveness model from a US health care perspective combining a decision tree and Markov trace. The DAWN and DEFUSE 3 trials enrolled 206 international patients from 2014 to 2017 and 182 US patients from 2016 to 2017, respectively. Patients were followed until 3 months after stroke. The clinical outcome at 3 months was available for 29 subgroups of patients with AIS and anterior circulation large vessel occlusions. Data analysis was conducted from July 2018 to October 2019.

**Exposures:**

MT with SMC in the extended treatment window vs SMC alone.

**Main Outcomes and Measures:**

Expected costs and quality-adjusted life-years (QALYs) during lifetime were estimated. Deterministic results (incremental costs and effectiveness, incremental cost-effectiveness ratios, and net monetary benefit) were presented, and probabilistic analyses were performed for the total populations and 27 patient subgroups.

**Results:**

In the DAWN study, the MT group had a mean (SD) age of 69.4 (14.1) years and 42 of 107 (39.3%) were men, and the control group had a mean (SD) age of 70.7 (13.2) years and 51 of 99 (51.5%) were men. In the DEFUSE 3 study, the MT group had a median (interquartile range) age of 70 (59-79) years, and 46 of 92 (50.0%) were men, and the control group had a median (interquartile range) age of 71 (59-80) years, and 44 of 90 (48.9%) were men. For the total trial population, incremental cost-effectiveness ratios were $662/QALY and $13 877/QALY based on the DAWN and DEFUSE 3 trials, respectively. MT with SMC beyond 6 hours had a probability greater than 99.9% of being cost-effective vs SMC alone at a willingness-to-pay threshold of $100 000/QALY. Subgroup analyses showed a wide range of probabilities for MT with SMC to be cost-effective at a willingness-to-pay threshold of $50 000/QALY, with the greatest uncertainty observed for patients with a National Institute of Health Stroke Scale score of at least 16 and for those aged 80 years or older.

**Conclusions and Relevance:**

The results of this study suggest that late MT added to SMC is cost-effective in all subgroups evaluated in the DAWN and DEFUSE 3 trials, with most results being robust in probabilistic sensitivity analyses. Future MT evidence-gathering could focus on older patients and those with National Institute of Health Stroke Scale scores of 16 and greater.

## Key Points

**Question:**

Is mechanical thrombectomy in the extended treatment window cost-effective across patient subgroups in the United States?

**Findings:**

This economic evaluation study found that mechanical thrombectomy provides good value for money in all the defined subgroups the 2 randomized clinical trials evaluated. Sensitivity analyses revealed a wide range of probabilities for late mechanical thrombectomy to be cost-effective at the willingness-to-pay threshold of $50 000 per quality-adjusted life-year.

**Meaning:**

The results of this study suggest that attention should be placed on increasing access to mechanical thrombectomy rather than on developing subgroup-specific guidelines unless workforce and budget constraints require prioritization.

## Teaser

This economic evaluation study compares the cost-effectiveness of 2 strategies (mechanical thrombectomy with standard medical care vs standard medical care alone) for various subgroups of patients with acute ischemic stroke who were receiving care more than 6 hours after symptom onset.

## Introduction

The randomized clinical trials DAWN and DEFUSE 3 demonstrated superior functional outcomes of mechanical thrombectomy (MT) at 90 days among patients with acute ischemic stroke (AIS) treated 6 to 24 hours after they were last known well (eAppendix in the Supplement).^1,2^ Health-economic evidence is needed to determine whether the short-term functional benefit of late MT translates to cost-effectiveness in the United States over a lifetime. A prolonged MT window implies advanced neuroimaging selection of patients and greater neurology and endovascular staff, which are costly and potentially critical resources. Furthermore, factors such as time from symptom onset, patient characteristics, National Institutes of Health Stroke Scale (NIHSS) score, mode of presentation, imaging criteria, and localization of the occlusion might influence the long-term value of late MT. Analyzing the magnitude of the long-term cost-effectiveness of late window MT per patient subgroup could expand the evidence and help inform allocation of critical resources. The aim of this study was to compare the cost-effectiveness of MT with standard medical care (SMC) vs SMC alone by patient subgroup in the late window in the United States.

## Methods

### Study Design

We framed, structured, populated, and dealt with uncertainty according to the formal steps of cost-effectiveness modeling.^3,4^ A short-run decision tree model (3-month time horizon) and a lifetime Markov state-transition model were designed in Microsoft Excel version 2002 to analyze and compare the costs and outcomes of 2 care pathways, ie, MT with SMC vs SMC alone, in patients with AIS 6 to 24 hours after symptom onset in the United States. We defined SMC as antiplatelet therapy and supportive care according to local guidelines. Subgroup analyses were performed based on the subgroup data published for the 2 trials.^1,2^ A hypothetical US cohort of 1000 patients with AIS was modeled using the same age characteristics and criteria as defined in the trials.

The efficacy data from the 2 trials were used as 3-month input parameters in our short-run model. Other input parameter values, such as costs, utilities, and transition probabilities, were drawn from the literature. Costs and quality-adjusted life-years (QALYs) were calculated for each care strategy for a lifetime time horizon. Costs and outcomes were discounted at 3% annually, and the US health care perspective was used. Per our institutional policy, ethical approval is not required for this study type. This study followed the Consolidated Health Economic Evaluation Reporting Standards (CHEERS) reporting guideline.^5^

### Model Structure

#### Decision Tree Model

**Figure 1.**
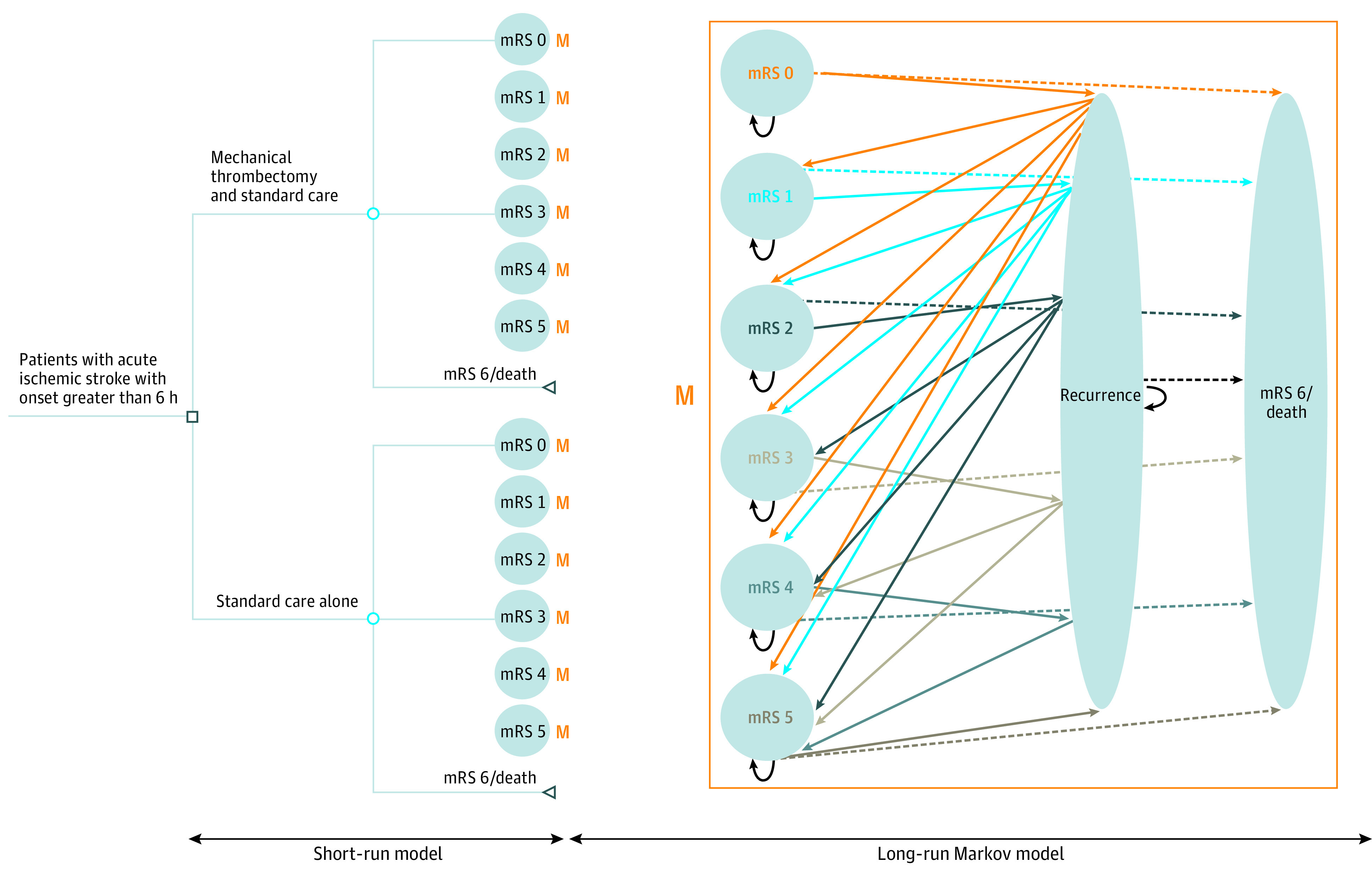
Model Structure Representing the 3-Month Acute Phase and the Long-term Sequence of Events Post-initial Stroke Patient’s entry into the model occurs at presentation with acute ischemic stroke between 6 and 24 hours after stroke onset. Patients receive either mechanical thrombectomy with standard care or standard care alone as acute treatment and enter a health state of the modified Rankin Scale (mRS) after the 3-month acute phase. Patients who survive the initial acute phase enter the Markov model, which runs on 3-month cycles. After every 3 months, patients can remain in their current mRS state, experience a recurrent stroke, or die from a nonstroke-related cause. After a recurrent stroke, patients either die or transition to a worse mRS state (with risk equally divided among worse states).

We built a short-run decision model to estimate the costs and clinical outcomes at 90 days after the first AIS (Figure 1). Patients with AIS in a hypothetical cohort were distributed at 90 days into 1 of 7 possible modified Ranking Scale (mRS) scores.^6^ Treatment outcomes were assumed to occur during the acute phase. The probabilities for a patient to be allocated to the different mRS states at 90 days were obtained from the DAWN and DEFUSE 3 results for both the total study populations and for patient subgroups (eTable 1 in the Supplement). The group of patients in the combined mRS 5 and 6 group in the DAWN results was split into 2 groups (mRS 5 and mRS 6) according to the relative proportions of these 2 groups in the DEFUSE 3 trial. The mean age of the modeled cohort of AIS patients was customized based on the mean age per trial, per strategy (MT with SMC or SMC alone) and per patient subgroup. The ages modeled in our different analyses can be found in eTable 2 in the Supplement.

#### Markov Model

We included AIS patients who survived the initial 3-month acute phase in a long-run Markov state-transition model (Figure 1) built to estimate lifetime costs and health outcomes. The model was composed of 3-month cycles, which were repeated until all patients theoretically died to reflect a lifetime time horizon. Every 3 months, patients could remain in their current mRS state, experience a recurrent stroke, or die from nonstroke-related cause. Patients experiencing a recurrent stroke could either die or transition to a worse mRS state (with an equal risk of transitioning to a worse state). Because previous studies indicated an increased mortality for dependent patients (ie, patients with mRS 3, 4, or 5) compared with independent patients (ie, patients with mRS 0, 1, or 2),^7,8^ we used mRS state-specific hazard ratios (Table 1).^8,9,10,11,12,13,14,15,16,17,18,19^ We used US life tables for age-adjusted and sex-adjusted all-cause mortality rates applied from the end of month 3 onward.^20^

**Table 1.** List of Input Parameters^a^

Model input parameters	Base-case value	Distribution	Range	Source
**3-Monthly transition probabilities in the Markov model**				
State after recurrence of a patient in mRS 0				Fagan et al^9,10^ for probability of death and assumption that patient has an equal risk of transitioning to 1 of the worse states
mRS 1	0.19	Dirichlet	0-1	
mRS 2	0.19			
mRS 3	0.19			
mRS 4	0.19			
mRS 5	0.19			
mRS 6 or death	0.0513			
State after recurrence of a patient in mRS 1				
mRS 2	0.24	Dirichlet	0-1	
mRS 3	0.24			
mRS 4	0.24			
mRS 5	0.24			
mRS 6 or death	0.0513			
State after recurrence of a patient in mRS 2				
mRS 3	0.32	Dirichlet	0-1	
mRS 4	0.32			
mRS 5	0.32			
mRS 6 or death	0.0513			
State after recurrence of a patient in mRS 3				
mRS 4	0.47	Dirichlet	0-1	
mRS 5	0.47			
mRS 6 or death	0.0513			
State after recurrence of a patient in mRS 4		β	Parameters, α = 94.9; β = 5.1	
mRS 5	0.95			
mRS 6 or death	0.0513			
Death hazard ratios				
mRS 0	1	Log normal	SE, 0.076	Samsa et al,^8^ 1999
mRS 1	1		SE, 0.46	
mRS 2	1.11		SE, 0.46	
mRS 3	1.27		SE, 0.46	
mRS 4	1.71		SE, 0.46	
mRS 5	2.37		SE, 0.46	
Recurrence	0.013	β	Parameters, α = 13; β = 986	Ganesalingam et al,^11^ 2015
**Costs and resource use**				
Costs and resource use in the decision tree				
CT	$198	β Pert	$168-$228	CMS,^12^ 2015
CTA	$774	β Pert	$658-$890	
MRI	$625	β Pert	$531-$718	
MRA	$1023	β Pert	$870-$1176	
CTP	$836	β Pert	$711-$961	Jackson et al,^13^ 2010
Software	$89	β Pert	$44-$520	eTable 3 in the Supplement
Frequency of CTA vs MRA	0.5	Uniform	0-1	Assumed
Frequency of CTP vs MRI	0.5	Uniform	0-1	
IV-tPA acquisition and administration	$8004	β Pert	$6403-$9605	Kunz et al,^14^ 2016
MT devices, nonphysician room personnel, and operating room overhead	$15 836	β Pert	$5270-$26 401	Shireman et al,^15^ 2017
Physician costs	$2749	β Pert	$1262-$4236	
Acute first 3-mo costs				
mRS 0	$14 382	β Pert	$14 210-$14 554	Joo et al,^16^ 2017; no IV-tPA group, weighted mean of costs for the group aged 65-80 y and the group aged ≥80 y
mRS 1	$14 382	β Pert	$14 210-$14 554	
mRS 2	$14 382	β Pert	$14 210-$14 554	
mRS 3	$17 879	β Pert	$17 660-$18 097	
mRS 4	$17 879	β Pert	$17 660-$18 097	
mRS 5	$17 879	β Pert	$17 660-$18 097	
mRS 6 or death	$23 498	β Pert	$22 614-$24 382	
Costs and resource use in the Markov model				
3-monthly long-term health care costs, day 90 onward				
mRS 0	$2836	β Pert	$2269-$3403	Shireman et al,^15^ 2017; Earnshaw et al,^17^ 2009, for range
mRS 1	$2741	β Pert	$2336-$3504	
mRS 2	$3378	β Pert	$2703-$4054	
mRS 3	$5801	β Pert	$4641-$6961	
mRS 4	$11 742	β Pert	$9393-$14 090	
mRS 5	$17 262	β Pert	$13 809-$20 714	
Cost of recurrent stroke 90 d following stroke recurrence	Values for the total population	No independent distribution was defined; costs vary based on the 2000 PSA results (ie, expected value of costs) of decision tree	Based on the 95% CIs of the 2000 PSA results	From short-run 90-d decision tree
MT with SMC strategy				
DAWN trial	$37 974		$29 607-$47 008	
DEFUSE 3 trial	$38 500		$30 077-$47 738	
SMC alone strategy				
DAWN trial	$20 693		$20 073-$21 378	
DEFUSE 3 trial	$20 479		$19 834-$21 198	
**Utilities**				
mRS 0	0.85	β	0.80-1.00	Gage et al,^18^ 1998; Nelson et al,^19^ 2016; Earnshaw et al,^17^ 2009, for the range.
mRS 1	0.80	β	0.80-0.95	
mRS 2	0.70	β	0.68-0.90	
mRS 3	0.51	β	0.45-0.65	
mRS 4	0.30	β	0.10-0.40	
mRS 5	0.15	β	0-0.32	
mRS 6 or death	0	NA	NA	
Recurrent stroke 90 d following stroke recurrence	Values for the total population	No independent distribution was defined; utilities vary based on the 2000 PSA results (ie, expected value of utility) of the decision tree	Based on the 95% CI of the 2000 runs	From short-run 90-d decision-tree
MT with SMC strategy				
DAWN trial	0.49		0.43-0.56	
DEFUSE 3 trial	0.48		0.42-0.56	
SMC alone strategy				
DAWN trial	0.31		0.24-0.37	
DEFUSE 3 trial	0.31		0.24-0.37	

Abbreviations: CT, computed tomography; CTA, CT angiography; CTP, CT perfusion; IV-tPA, intravenous tissue plasminogen activator; MRA, magnetic resonance angiography; MRI, MR imaging; mRS, modified Ranking Scale; MT, mechanical thrombectomy; SMC, standard medical care.

^a^ Input parameters related to efficacy of MT with SMC and SMC alone, used in the decision tree, can be found in eTable 1 in the Supplement.

Patients experiencing a recurrent stroke were managed with the same treatment strategy (ie, MT with SMC or SMC only) as their initial treatment strategy. Based on previous studies, the risk of stroke recurrence was assumed to be equal across mRS states.^11,21^ We assumed that patients could experience only 1 recurrent stroke per 3-month cycle. The transition probabilities used in the Markov model can be found in Table 1.

### Costs and Resource Inputs

All costs were calculated in US dollars for the fiscal year 2019. We inflated costs originating from previous years based on the general Consumer Price Index.^22^ Costs and resources used in the model are presented in Table 1.

For SMC alone, patients were assumed to have received computed tomography (CT) and CT angiography (CTA). For MT with SMC, patients were assumed to have received CT and CTA or magnetic resonance angiography (MRA) and CT perfusion (CTP) or MR imaging (MRI). We assumed that CTA was used as often as MRA and that CTP was used as often as MRI. The cost of the software used to assess the infarct volume with MRI and CTP was added (eTable 3 in the Supplement). The cost of intravenous thrombolysis included acquisition and administration.^14^ This cost was included in both the MT with SMC and SMC strategies proportionally to the frequency of use in each group of the DAWN and DEFUSE 3 trials (eTable 4 in the Supplement). The cost of MT included the cost of the devices, nonphysician room personnel, and operating room overhead.^15^ The physician costs related to the delivery of MT were added.^15^

Based on the literature, the mean acute costs of the first 90 days after AIS and the mean 3-month long-term costs were dependent on the severity of the outcome (ie, on mRS state). The acute costs reflected the mean payment per patient with ischemic stroke older than 65 years discharged to home after hospitalization with an mRS score of less than 2, discharged to any destination except home with an mRS score of 3 to 5, and dying at the hospital with an mRS score of 6, based on original data from the 2010 to 2013 MarketScan Commercial Claims and Encounters Inpatient Database and Medicare Supplemental and Coordination of Benefits Database.^16,23^ Long-term mRS state-specific poststroke costs were based on observed data from a prospective economic study conducted alongside the SWIFT-PRIME trial.^24^ The long-term costs were based on Medicare inpatient and outpatient claims 3 months after the initial hospitalization and until death for 958 patients treated in 2 stroke centers in the United-States between 2010 and 2014.^15^ Nursing home costs were included. The cost of a recurrent stroke was derived from the findings of the decision tree and assumed to be specific to the MT with SMC strategy or SMC strategy alone. As such, it represents the cost estimate to identify and treat a typical AIS according to the strategy defined in the decision tree.

### Utilities and Quality of Life

Utilities were assigned to each mRS state based on survey data from a large sample of individuals at increased risk of stroke using the time trade-off method to value hypothetical health states. We chose this because of its methods and its use in recent US cost-effectiveness models.^18,19^

Utility values (ranges) were defined as 0.85 (0.80-1.00) for mRS 0; 0.80 (0.80-0.95) for mRS 1; 0.70 (0.68-0.90) for mRS 2; 0.51 (0.45-0.65) for mRS 3; 0.30 (0.10 to 0.40) for mRS 4; and 0.15 (0-0.32) for mRS 5. The utility of a recurrent stroke was assumed to be specific to each pathway and derived from the outcomes of the short-run model. Utilities were varied according to a β distribution (Table 1).

### Subgroup Analyses

The published results of the DAWN and DEFUSE 3 trials allowed for 29 subgroup analyses. The mean ages reported for the total study population from the trials (control and intervention groups) were used by default except for subgroups defined by age (eTable 2 in the Supplement). The sample size for each subgroup was modeled according to the trial subgroups (eTable 1 in the Supplement). Cost-effectiveness analyses were conducted for the total study populations and patient subgroups defined by time from stroke onset, age, NIHSS score, mode of presentation, clinical infarct mismatch (group A, aged ≥80 years, NIHSS score ≥10, and infarct volume <21 mL; group B, aged <80 years, NIHSS score ≥10, and infarct volume <31 mL; group C, aged <80 years, NIHSS score ≥20, and infarct volume 31-51 mL), occlusion location, time of symptom first observed, and trial eligibility criteria.

### Statistical Analysis

No statistical tests were conducted. No hypothesis testing nor level of statistical significance was relevant to our analysis. We estimated the credibility intervals (CI) surrounding the mean values when relevant (Table 2).

**Table 2.** Expected Values of Cost-effectiveness of Mechanical Thrombectomy With Standard Medical Care vs Standard Medical Care Alone per Patient with Acute Ischemic Stroke Stratified by Subgroup in the Base Case

Subgroups according to criteria	DAWN				DEFUSE 3			
	Incremental costs (95% CI), $	Incremental QALY (95% CI)	ICER ($/QALY)	Incremental NMB (95% CI), $^a^	Incremental costs (95% CI), $	Incremental QALY (95% CI)	ICER ($/QALY)	NMB (95% CI), $^a^
Time from stroke onset, h^b^								
6 to ≤24, ie, full DAWN group	1380 (−62 510 to 52 675)	2.085 (1.283 to 3.239)	662	207 125 (121 419 to 328 519)	NA	NA	NA	NA
6 to ≤12	−24 340 (−109 596 to 51 786)	1.968 (0.793 to 3.403)	−12 369 (dominant)	221 130 (87 319 to 389 968)	NA	NA	NA	NA
>12	23 446 (−46 225 to 85 923)	2.244 (1.259 to 3.468)	10 063	200 996 (88 012 to 340 344)	NA	NA	NA	NA
6 to ≤16, ie, full DEFUSE 3 group	NA	NA	NA	NA	25 098 (−35 502 to 76 710)	1.809 (0.972 to 2.847)	13 877	155 767 (63 411 to 277 583)
6 to ≤11	NA	NA	NA	NA	8511 (−62 240 to 71 472)	1.086 (−0.036 to 2.387)	7838	100 076 (−26 195 to 247 802)
>11	NA	NA	NA	NA	49 256 (−32 490 to 128 731)	2.599 (1.453 to 4.016)	18 951	210 654 (85 444 to 366 389)
Age, y								
≥80	14 451 (−38 704 to 58 813)	0.677 (−0.110 to 1.666)	19 994	57 825 (−23 371 to 153 740)	10 957 (−45 627 to 59 194)	0.504 (−0.256 to 1.376)	21 733	39 461 (−48 722 to 198 257)
<80	−28 675 (−106 405 to 36 384)	2.062 (1.043 to 3.344)	−13 908 (dominant)	234 857 (114 005 to 388 901)	29 235 (−40 244 to 89 664)	1.930 (0.906 to 3.214)	15 151	163 727 (45 800 to 314 595)
NIHSS score								
10 to <17	−14 030 (−91 236 to 47 696)	2.4972 (1.331 to 3.932)	−5675 (dominant)	261 266 (129 596 to 424 608)	NA	NA	NA	NA
≥17	18 125 (−62 348 to 81 368)	1.427 (0.472 to 2.581)	12 698	124 620 (24 100 to 258 074)	NA	NA	NA	NA
<16	NA	NA	NA	NA	5473 (−62 186 to 61 355)	1.540 (0.363 to 2.871)	3555	148 488 (10 890 to 304 083)
≥16	NA	NA	NA	NA	56 866 (−21 042 to 132 049)	1.334 (0.256 to 2.443)	42 635	76 514 (−42 001 to 196 691)
Mode of presentation								
Wake up	−9275 (−88 519 to 58 881)	2.241 (1.172 to 3.549)	−4139 (dominant)	233 341 (119 290 to 386 019)	10 318 (−81 241 to 82 859)	1.949 (0.891 to 3.355)	5294	184 593 (73 893 to 338 877)
Witnessed	13 005 (−117 373 to 130 783)	2.921 (0.786 to 5.218)	4453	279 067 (36 291 to 537 288)	21 061 (−57 981 to 93 981)	2.143 (0.721 to 3.738)	9828	193 230 (24 396 to 377 276)
Unwitnessed	9522 (−86 403 to 95 731)	1.507 (0.094 to 3.044)	6319	141 170 (−12 913 to 312 454)	NA	NA	NA	NA
Clinical infarct mismatch^c^								
Group A	14 451 (−35 197 to 56 392)	0.723 (−0.022 to 1.623)	19 994	57 825 (−20 168 to 153 024)	NA	NA	NA	NA
Group B	−28 621 (−106 109 to 37 741)	1.988 (0.95 to 3.348)	−14 397 (dominant)	227 428 (106 248 to 387 532)	NA	NA	NA	NA
Group C	−22 379 (−214 699 to 145 798)	1.380 (−1.639 to 4.234)	−16 211 (dominant)	160 340 (−153 172 to 480 987)	NA	NA	NA	NA
Occlusion location								
ICA	22 813 (−80 872 to 118 427)	1.930 (0.149 to 4.018)	11 819	170 202 (−19 862 to 383 228)	NA	NA	NA	NA
MCA M1	804 (−66 161 to 56 198)	1.982 (1.097 to 3.162)	406	197 363 (91 793– 330 057)	NA	NA	NA	NA
MCA M2	−49 769 (−283 278 to 189 815)	1.782 (−3.319 to 6.476)	−27 934 (dominant)	227 933 (−303 565 to 711 711)				
Time from symptom first observed, h^d^								
≤6	9340 (−61 634 to 67 879)	2.006 (1.083 to 3.245)	4657	191 216 (81 783 to 334 672)	NA	NA	NA	NA
>6	−18 671 (−115 297 to 60 878)	2.367 (1.01 to 3.96)	−7888 (dominant)	255 379 (104 956 to 429 484)	NA	NA	NA	NA
Trial eligibility criteria								
Not DAWN eligible	NA	NA	NA	NA	46 853 (−34 763 to 119 181)	1.972 (0.577 to 3.602)	23 763	150 317 (−4487 to 326 266)
DAWN eligible	NA	NA	NA	NA	11 420 (−59 575 to 68 490)	1.589 (0.622 to 2.778)	7186	147 497 (36 712 to 282 645)

Abbreviations: ICA, internal carotid artery; ICER, incremental cost-effectiveness ratio; MCA, middle cerebral artery; NA, not applicable; NIHSS, National Institutes of Health Stroke Scale; NMB, net monetary benefit; QALY, quality-adjusted life-year.

^a^ NMB set at a willingness-to-pay threshold of $100 000/QALY.

^b^ Time from stroke onset to randomization. Data from the DAWN trial were used to estimate the cost-effectiveness of mechanical thrombectomy with standard medical care vs standard medical care alone. These results are presented for patients treated between 6 and 24 hours, between 6 and 12 hours, and between 12 and 24 hours from stroke onset. Data from the DEFUSE 3 trial were used to estimate the cost-effectiveness of mechanical thrombectomy with standard medical care vs standard medical care alone for patients treated between 6 and 16 hours, between 6 and 11 hours, and between 11 and 16 hours from stroke onset. The same logic applies to the other subgroups per trial defined according to the different criteria presented in the first column.

^c^ Clinical infarct mismatch indicates a mismatch between the severity of the clinical deficit and the infarct volume defined according to the following groups: A, aged 80 years and older, NIHSS score of at least 10, and infarct volume of less than 21 mL; B, younger than 80 years, NIHSS score of at least 10, and infarct volume of less than 31 mL; C, younger than 80 years, NIHSS score of at least 20, and infarct volume between 31 and 51 mL.

^d^ Time of symptom first observed to randomization.

We performed a probabilistic sensitivity analysis (PSA) in Excel to assess how parameter uncertainty affected the cost-effectiveness results. In this process, we assigned a distribution to each parameter according to the level of uncertainty regarding its deterministic value. A random value was drawn from each distribution, and the set of drawn values was used to calculate the results of interest. This process was repeated in 2000 simulations to generate 2000 estimates of the costs and QALYs for each strategy. The proportion of simulations when MT with SMC had a higher net monetary benefit (NMB) than SMC alone was calculated for different values of the willingness-to-pay (WTP) threshold for a QALY. The results were described using cost-effectiveness acceptability curves, in which each curve represented the probability that MT with SMC was cost-effective compared with SMC alone at different WTP thresholds.

## Results

In the DAWN study, the MT group had a mean (SD) age of 69.4 (14.1) years and 42 of 107 (39.3%) were men, and the control group had a mean (SD) age of 70.7 (13.2) years and 51 of 99 (51.5%) were men. In the DEFUSE 3 study, the MT group had a median (interquartile range) age of 70 (59-79) years, and 46 of 92 (50.0%) were men, and the control group had a median (interquartile range) age of 71 (59-80) years, and 44 of 90 (48.9%) were men.

Table 2 shows the base-case cost-effectiveness results of MT with SMC vs SMC alone per trial inputs. Based on the total population from either trial, MT with SMC generated higher costs and more QALYs compared with SMC alone. The resulting incremental cost-effectiveness ratios (ICERs) were $662/QALY and $13 877/QALY based on the DAWN and DEFUSE 3 trial inputs, respectively. The incremental costs and QALYs for the total populations and all subgroups are plotted in eFigure 1 in the Supplement. In all subgroups, MT with SMC led to better health outcomes than SMC alone. In 8 of 18 DAWN subgroups (44.4%), MT with SMC was cost saving and more effective (ie, dominant) compared with SMC alone. Based on the DEFUSE 3 trial results, $3555 was the minimum cost to gain 1 QALY and was observed in patients with baseline NIHSS scores of less than 16. The maximum cost to gain 1 QALY was $19 994, based on the DAWN results and observed for patients older than 80 years and those in clinical infarct mismatch group A. Based on the DEFUSE 3 results, the maximum cost to gain 1 QALY was $42 635 for patients with baseline NIHSS score of 16 or greater (Table 1).

**Figure 2.**
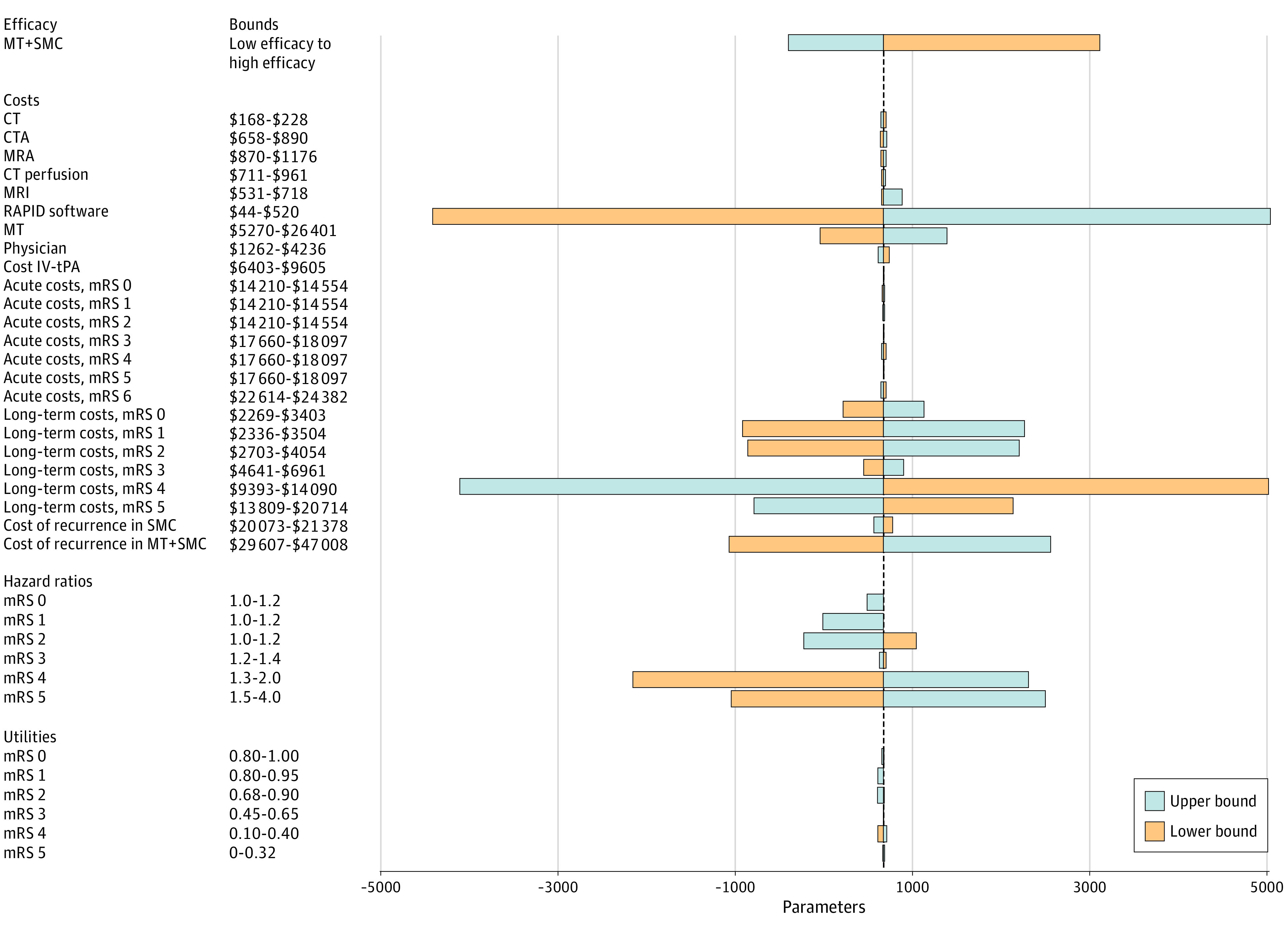
One-way Sensitivity Analysis Based on the DAWN Inputs Deterministic 1-way sensitivity analysis of model input parameters grouped by categories based on the DAWN inputs. The plot shows how varying input parameters to the limits reported, 1 at a time, affects the incremental cost-effectiveness ratio (ICER), while keeping all the other model input parameters at their base-case value. The orange bars represent how the lower bounds affect the ICER, and the blue bars represent how the upper bounds affect the ICER. The lengths of the bars reveals the degree of influence that 1 input parameter has on the ICER compared with the base-case ICER of $662 per quality-adjusted life-years. Low and high efficacy of mechanical thrombectomy with standard medical care (MT+SMC) was defined by the following distribution of patients on the modified Rankin Scale (mRS) at 3 months: low efficacy, mRS 0, 7%; mRS 1, 20%; mRS 2, 15%; mrS 3, 14%; mRS 4, 14.5%; mRS 5, 11.5%; and mRS 6, 18%; high efficacy, mRS 0, 10%; mRS 1, 23%; mRS 2, 18%; mRS 3, 13%; mRS 4, 12%; mRS 5, 9%; and mRS 6, 15%. CT indicates computed tomography; CTA, CT angiography; IV-tPA, intravenous tissue plasminogen activator; MRA, magnetic resonance angiography; and MRI, MR imaging.

Figure 2 presents the results of the deterministic 1-way sensitivity analysis based on the DAWN inputs. The ICER is particularly sensitive to the cost of MT. Additionally, an increase in the long-term cost of mRS 4 and 5 led to a more favorable ICER. The same analysis based on the DEFUSE 3 inputs led to similar results (eFigure 2 in the Supplement).

**Figure 3.**
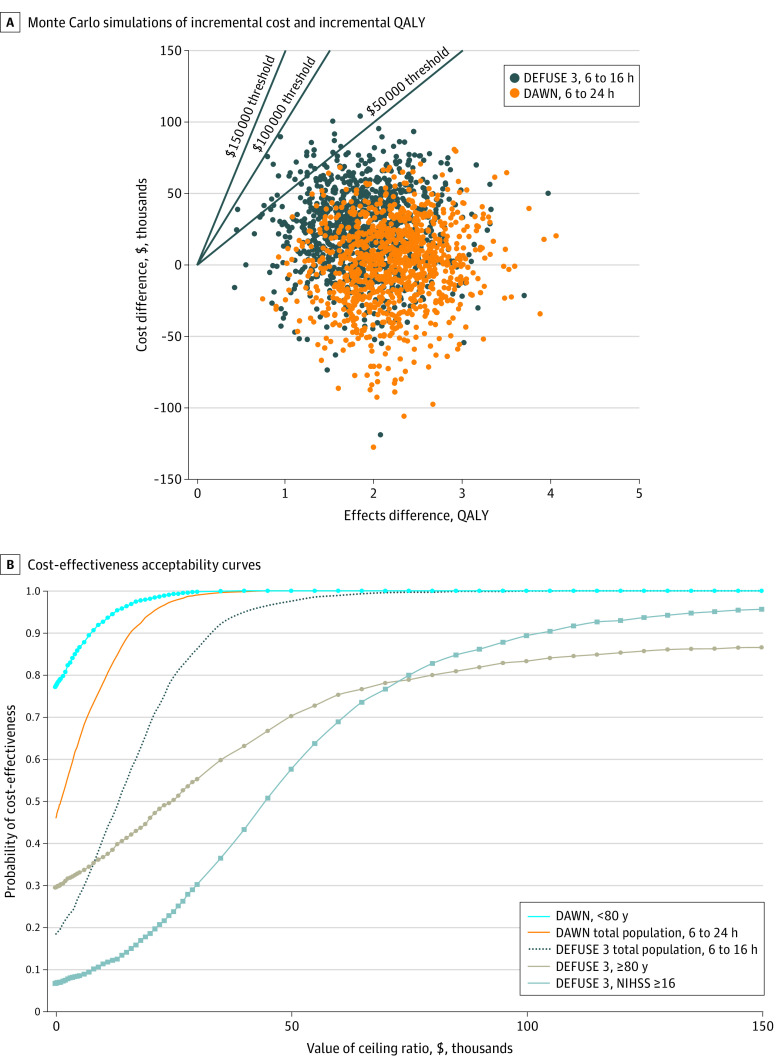
Monte Carlo Simulations of Incremental Cost and Incremental Quality-Adjusted Life-Years (QALY) of Mechanical Thrombectomy With Standard Medical Care for the Full Population A, The results are shown as scatterplots of incremental costs and incremental QALYs of mechanical thrombectomy with standard medical care vs standard medical care alone per patient with acute ischemic stroke for the full population per trial. Each dot represents 1 simulation run. The black lines indicate 3 different willingness-to-pay thresholds per QALY. The number of dots below a specific line represent the probability for mechanical thrombectomy with standard medical care to be cost-effective at the related WTP threshold. B, Each curve shows the probability that mechanical thrombectomy with standard medical care is cost-effective at different values of willingness to pay for a QALY for the full population and different subgroups. Curves for the SMC alone strategy are not shown.

The uncertainty surrounding the base-case estimates for the total population per trial is shown in Figure 3A. The PSA demonstrated that MT with SMC had either a 100% (based on the DAWN results) or a 99.9% (based on the DEFUSE 3 results) probability of being cost-effective at the WTP threshold of $100 000 per QALY. At a threshold of $50 000 per QALY, the probability of MT with SMC to be cost-effective was 100% and 97.5% based on the DAWN and DEFUSE 3 results, respectively. Scatter plots of incremental costs and incremental QALYs for all subgroups per trial can be found in eTable 5 in the Supplement.

Figure 3B shows the probability that MT with SMC is cost-effective at different WTP thresholds for a QALY for the total populations and the 3 subgroups characterized by the most extreme results. At the threshold of $100 000/QALY, the probability for MT with SMC to be cost-effective was among the 2 lowest for patients aged 80 years or older (DEFUSE 3) (83.3%). At this threshold, the lowest probability (79.8%) was observed for patients with a middle cerebral artery M2 occlusion (DAWN), but given the small sample size for this group, its curve is reported only in eFigure 3 in the Supplement. At $50 000/QALY, the probability for MT with SMC to be cost-effective was less than 60% for patients with a baseline NIHSS score of 16 or greater in the DEFUSE 3 trial. At a low or no WTP for a QALY, the probability of MT with SMC being cost-effective was the highest among patients younger than 80 years (DAWN). eFigure 3 in the Supplement presents the consolidated results for all groups and subgroups.

## Discussion

Our main finding was that MT with SMC, compared with SMC alone, for patients with AIS and anterior large vessel occlusion in the late window is cost-effective in the United States. We performed model-based cost-effectiveness analyses of MT with SMC compared with SMC alone based on the DAWN and DEFUSE 3 trial results and found that MT with SMC met conventional standards for cost-effectiveness in all subgroups. Based on the American College of Cardiology and American Heart Association (ACC/AHA) policy statement defining cost-effectiveness levels to inform value-based policies, ICERs below $50 000/QALY suggest high-value care, while ICERs between $50 000/QALY and $150 000 suggest intermediate value.^25^ All the point estimates in the various subgroups suggest that MT with SMC provides high-value care (per the ACC/AHA standard) compared with SMC alone. The PSA results indicated that the minimum probability for MT with SMC to be cost-effective was approximately 80% at a threshold of $100 000/QALY across subgroups. Increased uncertainty regarding whether MT with SMC was cost-effective at $50 000/QALY appeared among patients with NIHSS scores of 16 or greater in the DEFUSE 3 trial and patients aged 80 years and older in both trials.

Our findings are consistent with previous studies but also push the boundary of knowledge regarding the cost-effectiveness of late MT. Kunz et al^14^ performed a model-based cost-effectiveness analysis of MT with SMC vs SMC in the early treatment window. Their calculated ICER ($3110/QALY) was similar to our findings in the late treatment window ($662/QALY and $13 877/QALY for the DAWN and DEFUSE 3 trials, respectively). Although their analysis was limited by the number of subgroups, their results were robust in most patient profiles. In another analysis in the early window, Kunz et al^26^ found that MT with SMC was cost-effective in all age groups analyzed (ie, 50-100 years at stroke onset), with increased cost-effectiveness observed in younger patients, which is also consistent with our findings. In contrast, Pizzo et al^27^ and Peultier et al^28^ demonstrated that MT with SMC vs SMC alone was cost-effective in the late window in the United Kingdom. However, by targeting the UK market, their studies may not be generalizable to the US cost structure and did not include subgroups.

Given that they demonstrated the cost-effectiveness of MT across all clinical subgroups, our findings have latent policy and clinical implications. Acute stroke treatment guidelines and quality measures should focus on increasing access to MT for all eligible US patients rather than on tailoring policies that prioritize specific subgroups. Specifically, policies are needed to improve stroke recognition and transportation to comprehensive stroke centers (providing MT) in light of the cost-effectiveness of MT, which does not depreciate significantly by stroke severity or age. Should additional MT trials be conducted, our results suggest potential value in reducing the uncertainty regarding the cost-effectiveness of MT in certain subgroups (ie, patients with NIHSS scores of 16 or greater and those aged 80 years and older). However, this is only important to improve the certainty that MT represents high-value care (ie, ICER <$50 000/QALY), as opposed to MT being cost-effective at the conventional thresholds of $100 000/QALY to $150 000/QALY used in the United States.^29^

Beyond the cost-effectiveness considerations, the evidence regarding the clinical effectiveness of MT in the extended time window presents challenges for fast and widespread implementation. Complex and transversal care by ambulance or air and personnel in emergency, neurology, radiology, and neuro-intervention might sometimes be limited and might not guarantee access to MT for all eligible patients. Local and national policies to increase staffing in these professions may be necessary to meet this burgeoning clinical demand. Another short-term way to address potential critical limitations might be to prioritize the delivery of MT according to the degree and certainty of cost-effectiveness per patient subgroup.

Finally, in a country characterized by regions with low population density and large medically underserved areas and many individuals at increased risk of cardiovascular diseases, the delivery of MT in the late window might face organizational challenges.^30^ Access to MT for remote patients will probably require more investments in systems such as telemedicine and infrastructures both within and between states. Given that air transportation of patients will decrease time to treatment but increase costs, the optimal organization of stroke care will need to be investigated.

### Limitations

This study has limitations. First, our analyses are limited by the sample sizes of each subgroup included in the trials. However, we included sample size when estimating the probability of cost-effectiveness and found that there was a high probability that MT with SMC was cost-effective for most subgroups. Second, our analyses were limited by the selections of subgroups reported in the DAWN and DEFUSE 3 trial results. Thus, it is possible that MT with SMC might not be cost-effective in other subgroups. Third, while the DAWN trial included patients from multiple countries, we performed our analyses for the US setting. However, the DEFUSE 3 trial was restricted to 38 stroke centers in the United States, and our findings did not substantially differ between the 2 trials. Fourth, the quality-of-life estimates that we used were obtained from a study from 1998. However, these estimates have been used in recent studies. Fifth, although the cost of acquisition of software was included, it is important to highlight that this cost will depend on the number of patients diagnosed per facility per year. Further research might be needed to assess the cost-effectiveness of MT in the extended window at hospital level. Sixth, given the specifics (including the high costs) of the health care system in the United States, these results are not generalizable to other health care settings, where late MT might be more or less cost-effective.

## Conclusions

In conclusion, MT with SMC was generally cost-effective in all the subgroups evaluated in the DAWN and DEFUSE 3 trials. Future MT evidence-gathering is needed with a focus on older ages (ie, ≥80 years) and NIHSS scores of 16 and higher to reduce the uncertainties regarding these findings. More attention should be placed on increasing access to MT rather than on developing subgroup specific guidelines, unless workforce and budget constraints require prioritization.

## Notes

Supplement. **eAppendix. **Source Data **eFigure 1. **Scatterplot of Incremental Costs and Incremental QALYs per Subgroup and Trial **eFigure 2. **One-way Sensitivity Analysis Based on the DEFUSE 3 results **eFigure 3. **PSA Results, Cost-effectiveness Acceptability Curves Showing the Probability of MT With SMC Being Cost-effective at Different Values of WTP for a QALY, by Subgroups **eTable 1. **Short-run Model Input Parameters: Distribution of Patients on the mRS Scale at 3 Months After Initial AIS per Subgroup and Strategy per Trial **eTable 2. **Reported Age of Randomized Patients in the DAWN and DEFUSE 3 Trials and Parameterized Age of Patients in our Model **eTable 3. **Calculation Methods for the Cost of Software per Ischemic Stroke Patient **eTable 4.** Reported Frequencies of Use of Intravenous Thrombolysis in the DAWN and DEFUSE 3 Trials **eTable 5. **PSA Results, Monte Carlo Simulations of Incremental Cost and Incremental QALY per Patient with patient of MT With SMC vs SMC alone **eReferences.**

## References

[1] Nogueira RG, Jadhav AP, Haussen DC, ; DAWN Trial Investigators Thrombectomy 6 to 24 hours after stroke with a mismatch between deficit and infarct. N Engl J Med. 2018;378(1):11-21. doi:10.1056/NEJMoa1706442 29129157

[2] Albers GW, Marks MP, Kemp S, ; DEFUSE 3 Investigators Thrombectomy for stroke at 6 to 16 hours with selection by perfusion imaging. N Engl J Med. 2018;378(8):708-718. doi:10.1056/NEJMoa1713973 29364767 PMC6590673

[3] Drummond M, Sculpher M, Claxton K, Stoddart G, Torrance G Methods for the Economic Evaluation of Health Care Programmes. 4th ed Oxford University Press; 2015.

[4] Caro JJ, Briggs AH, Siebert U, Kuntz KM; ISPOR-SMDM Modeling Good Research Practices Task Force Modeling good research practices—overview: a report of the ISPOR-SMDM Modeling Good Research Practices Task Force-1. Med Decis Making. 2012;32(5):667-677. doi:10.1177/0272989X12454577 22990082

[5] Husereau D, Drummond M, Petrou S, ; CHEERS Task Force Consolidated Health Economic Evaluation Reporting Standards (CHEERS) statement. BMJ. 2013;346:f1049. doi:10.1136/bmj.f1049 23529982

[6] van Swieten JC, Koudstaal PJ, Visser MC, Schouten HJA, van Gijn J Interobserver agreement for the assessment of handicap in stroke patients. Stroke. 1988;19(5):604-607. doi:10.1161/01.STR.19.5.604 3363593

[7] Boudreau DM, Guzauskas GF, Chen E, Cost-effectiveness of recombinant tissue-type plasminogen activator within 3 hours of acute ischemic stroke: current evidence. Stroke. 2014;45(10):3032-3039. doi:10.1161/STROKEAHA.114.005852 25190439

[8] Samsa GP, Reutter RA, Parmigiani G, Performing cost-effectiveness analysis by integrating randomized trial data with a comprehensive decision model: application to treatment of acute ischemic stroke. J Clin Epidemiol. 1999;52(3):259-271. doi:10.1016/S0895-4356(98)00151-6 10210244

[9] Tung CE, Win SS, Lansberg MG Cost-effectiveness of tissue-type plasminogen activator in the 3- to 4.5-hour time window for acute ischemic stroke. Stroke. 2011;42(8):2257-2262. doi:10.1161/STROKEAHA.111.615682 21719767 PMC3164239

[10] Fagan SC, Morgenstern LB, Petitta A, Cost-effectiveness of tissue plasminogen activator for acute ischemic stroke: NINDS rt-PA Stroke Study Group. Neurology. 1998;50(4):883-890. doi:10.1212/WNL.50.4.883 9566367

[11] Ganesalingam J, Pizzo E, Morris S, Sunderland T, Ames D, Lobotesis K Cost-utility analysis of mechanical thrombectomy using stent retrievers in acute ischemic stroke. Stroke. 2015;46(9):2591-2598. doi:10.1161/STROKEAHA.115.009396 26251241 PMC4542565

[12] Centers for Medicare & Medicaid Services Physician fee schedule. Accessed July 29, 2020. https://www.cms.gov/Medicare/Medicare-Fee-for-Service-Payment/PhysicianFeeSched/PFS-Relative-Value-Files-Items/RVU15C

[13] Jackson D, Earnshaw SR, Farkouh R, Schwamm L Cost-effectiveness of CT perfusion for selecting patients for intravenous thrombolysis: a US hospital perspective. AJNR Am J Neuroradiol. 2010;31(9):1669-1674. doi:10.3174/ajnr.A2138 20538823 PMC7965001

[14] Kunz WG, Hunink MG, Sommer WH, Cost-effectiveness of endovascular stroke therapy: a patient subgroup analysis from a US healthcare perspective. Stroke. 2016;47(11):2797-2804. doi:10.1161/STROKEAHA.116.014147 27758942

[15] Shireman TI, Wang K, Saver JL, ; SWIFT-PRIME Investigators Cost-effectiveness of solitaire stent retriever thrombectomy for acute ischemic stroke: results from the SWIFT-PRIME Trial (Solitaire With the Intention for Thrombectomy as Primary Endovascular Treatment for Acute Ischemic Stroke). Stroke. 2017;48(2):379-387. doi:10.1161/STROKEAHA.116.014735 28028150 PMC5461963

[16] Joo H, Wang G, George MG Age-specific cost effectiveness of using intravenous recombinant tissue plasminogen activator for treating acute ischemic stroke. Am J Prev Med. 2017;53(6S2)(6 Suppl 2):S205-S212. doi:10.1016/j.amepre.2017.06.004 29153122 PMC5819005

[17] Earnshaw SR, Jackson D, Farkouh R, Schwamm L Cost-effectiveness of patient selection using penumbral-based MRI for intravenous thrombolysis. Stroke. 2009;40(5):1710-1720. doi:10.1161/STROKEAHA.108.540138 19286581

[18] Gage BF, Cardinalli AB, Owens DK Cost-effectiveness of preference-based antithrombotic therapy for patients with nonvalvular atrial fibrillation. Stroke. 1998;29(6):1083-1091. doi:10.1161/01.STR.29.6.1083 9626276

[19] Nelson RE, Okon N, Lesko AC, Majersik JJ, Bhatt A, Baraban E The cost-effectiveness of telestroke in the Pacific Northwest region of the USA. J Telemed Telecare. 2016;22(7):413-421. doi:10.1177/1357633X15613920 26541170

[20] Arias E, Xu J United States life tables, 2015. Natl Vital Stat Rep. 2018;67(7):1-64. Accessed March 15, 2019. https://www.cdc.gov/nchs/data/nvsr/nvsr67/nvsr67_07-508.pdf 30707669

[21] Guzauskas GF, Boudreau DM, Villa KF, Levine SR, Veenstra DL The cost-effectiveness of primary stroke centers for acute stroke care. Stroke. 2012;43(6):1617-1623. doi:10.1161/STROKEAHA.111.648238 22535277

[22] US Bureau of Labor Statistics. US city average Consumer Price Index, all items, 1982-84. Accessed March 15, 2019. https://www.bls.gov/regions/west/data/consumerpriceindex_us_table.pdf

[23] Truven Health Analytics. Truven Health MarketScan Research Databases (Commercial Claims and Encounters Medicare Supplemental: Data Year 2013 edition). Truven Health Analytics; 2014

[24] Saver JL, Goyal M, Bonafe A, ; SWIFT PRIME Investigators Stent-retriever thrombectomy after intravenous t-PA vs. t-PA alone in stroke. N Engl J Med. 2015;372(24):2285-2295. doi:10.1056/NEJMoa1415061 25882376

[25] Anderson JL, Heidenreich PA, Barnett PG, ; ACC/AHA Task Force on Performance Measures ACC/AHA statement on cost/value methodology in clinical practice guidelines and performance measures: a report of the American College of Cardiology/American Heart Association Task Force on Performance Measures and Task Force on Practice Guidelines. J Am Coll Cardiol. 2014;63(21):2304-2322. doi:10.1016/j.jacc.2014.03.016 24681044

[26] Kunz WG, Hunink MG, Dimitriadis K, Cost-effectiveness of endovascular therapy for acute ischemic stroke: a systematic review of the impact of patient age. Radiology. 2018;288(2):518-526. doi:10.1148/radiol.2018172886 29893641

[27] Pizzo E, Dumba M, Lobotesis K Cost-utility analysis of mechanical thrombectomy between 6 and 24 hours in acute ischemic stroke. Int J Stroke. 2020;15(1):75-84. doi:10.1177/1747493019830587 30758277

[28] Peultier AC, Redekop WK, Allen M, Peters J, Eker OF, Severens JL Exploring the cost-effectiveness of mechanical thrombectomy beyond 6 hours following advanced imaging in the United Kingdom. Stroke. 2019;50(11):3220-3227. doi:10.1161/STROKEAHA.119.026816 31637975 PMC6824506

[29] Neumann PJ, Cohen JT, Weinstein MC Updating cost-effectiveness—the curious resilience of the $50,000-per-QALY threshold. N Engl J Med. 2014;371(9):796-797. doi:10.1056/NEJMp1405158 25162885

[30] Havranek EP, Mujahid MS, Barr DA, ; American Heart Association Council on Quality of Care and Outcomes Research, Council on Epidemiology and Prevention, Council on Cardiovascular and Stroke Nursing, Council on Lifestyle and Cardiometabolic Health, and Stroke Council Social determinants of risk and outcomes for cardiovascular disease: a scientific statement from the American Heart Association. Circulation. 2015;132(9):873-898. doi:10.1161/CIR.0000000000000228 26240271

