# Supplementary material for: Cost-effectiveness of Mechanical Thrombectomy More Than 6 Hours After Symptom Onset Among Patients With Acute Ischemic Stroke
Source: JAMA Netw Open. 2020 Aug 25;3(8):e2012476. doi: 10.1001/jamanetworkopen.2020.12476 (PMC7448828; doi:10.1001/jamanetworkopen.2020.12476)
Supplement: Supplement. — eAppendix. Source Data eFigure 1. Scatterplot of Incremental Costs and Incremental QALYs per Subgroup and Trial eFigure 2. One-way Sensitivity Analysis Based on the DEFUSE 3 results eFigure 3. PSA Results, Cost-effectiveness Acceptability Curves Showing the Probability of MT With SMC Being Cost-effective at Different Values of WTP for a QALY, by Subgroups eTable 1. Short-run Model Input Parameters: Distribution of Patients on the mRS Scale at 3 Months After Initial AIS per Subgroup and Strategy per Trial eTable 2. Reported Age of Randomized Patients in the DAWN and DEFUSE 3 Trials and Parameterized Age of Patients in our Model eTable 3. Calculation Methods for the Cost of Software per Ischemic Stroke Patient eTable 4. Reported Frequencies of Use of Intravenous Thrombolysis in the DAWN and DEFUSE 3 Trials eTable 5. PSA Results, Monte Carlo Simulations of Incremental Cost and Incremental QALY per Patient with patient of MT With SMC vs SMC alone eReferences. [file jamanetwopen-3-e2012476-s001.pdf]

Supplementary Online Content

Peultier A-C, Pandya A, Sharma R, Severens JL, Redekop WK. Cost-effectiveness of mechanical thrombectomy more than 6 hours after symptom onset among patients with acute ischemic stroke. *JAMA Netw Open*. 2020;3(8):e2012476. doi:10.1001/jamanetworkopen.2020.12476

eAppendix. Source Data

eFigure 1. Scatterplot of Incremental Costs and Incremental QALYs per Subgroup and Trial

eFigure 2. One-way Sensitivity Analysis Based on the DEFUSE 3 results

eFigure 3. PSA Results, Cost-effectiveness Acceptability Curves Showing the Probability of MT With SMC Being Cost-effective at Different Values of WTP for a QALY, by Subgroups

eTable 1. Short-run Model Input Parameters: Distribution of Patients on the mRS Scale at 3 Months After Initial AIS per Subgroup and Strategy per Trial

eTable 2. Reported Age of Randomized Patients in the DAWN and DEFUSE 3 Trials and Parameterized Age of Patients in our Model

eTable 3. Calculation Methods for the Cost of Software per Ischemic Stroke Patient

eTable 4. Reported Frequencies of Use of Intravenous Thrombolysis in the DAWN and DEFUSE 3 Trials

eTable 5. PSA Results, Monte Carlo Simulations of Incremental Cost and Incremental QALY per Patient with patient of MT With SMC vs SMC alone

eReferences.

This supplementary material has been provided by the authors to give readers additional information about their work.

eAppendix. Source Data

In the DAWN trial, patients with an anterior circulation large vessel occlusion, NIHSS >10, and favorable imaging profiles were randomized to either MT (N=107) or medical management (N=99) between 6 to 24 hours after time last known well at centers in the United States, Canada, Europe, and Australia (9). In DEFUSE 3, patients with anterior circulation large vessel occlusions, NIHSS > 6, and favorable imaging profiles were randomized to MT (N=92) versus medical therapy (N=90) 6 to 16 hours after last known well. Patients were recruited from 38 centers located in the United States (10).

Informed consent was obtained from all participants or their legal representatives. MT was performed with the Trevo device by Stryker in DAWN and with any FDA-approved device in DEFUSE 3. Intravenous tissue plasminogen activator (IV-tPA) was allowed before randomization, if begun within 4.5 hours from symptom onset.

© 2020 Peultier A-C et al. *JAMA Network Open.*

eFigure 1. Scatterplot of Incremental Costs and Incremental QALYs per Subgroup and Trial

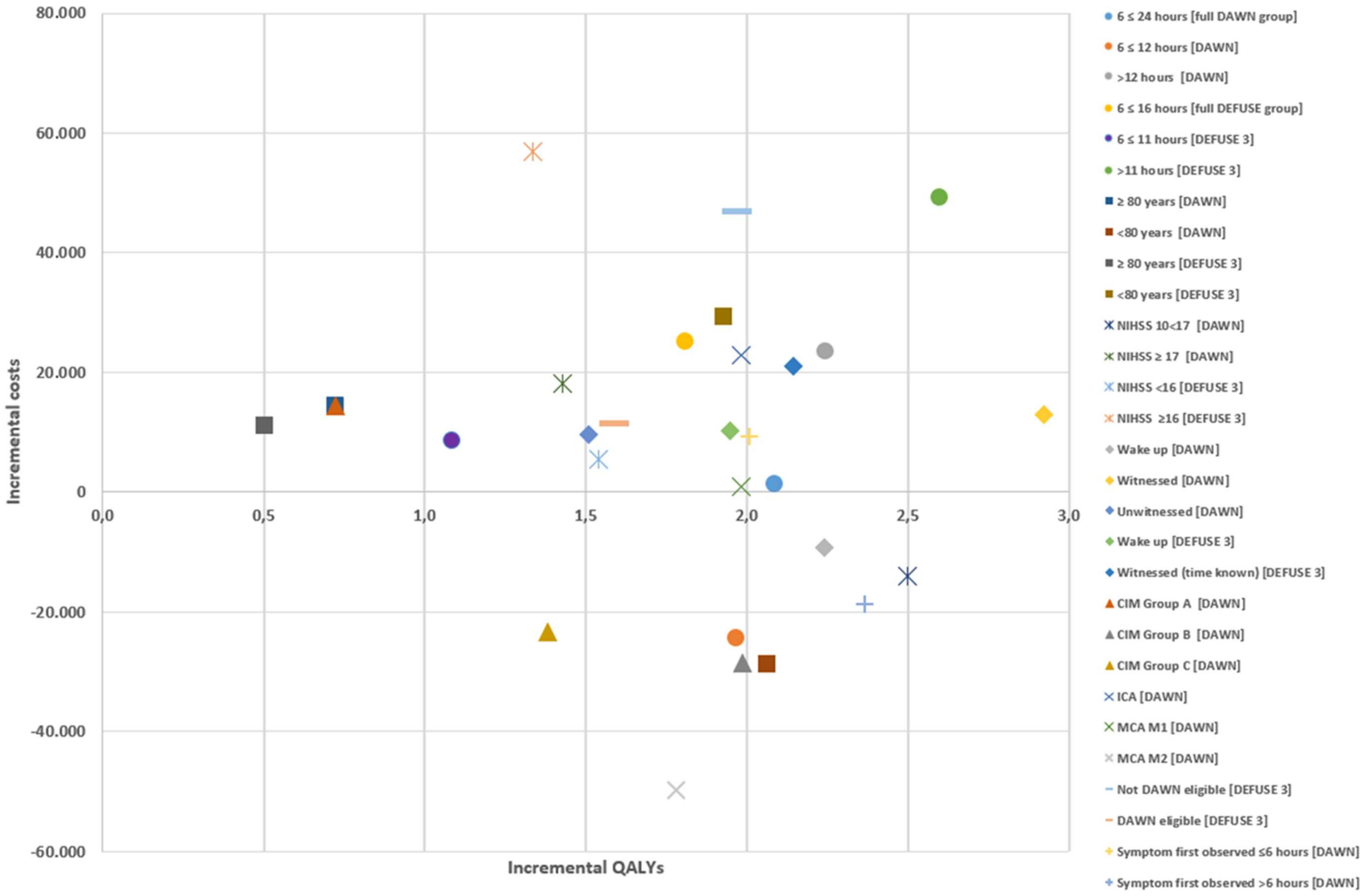

© 2020 Peultier A-C et al. *JAMA Network Open.*

eFigure 2. One-way Sensitivity Analysis Based on the DEFUSE 3 results

The plot shows ICER for MT+SMC versus SMC.

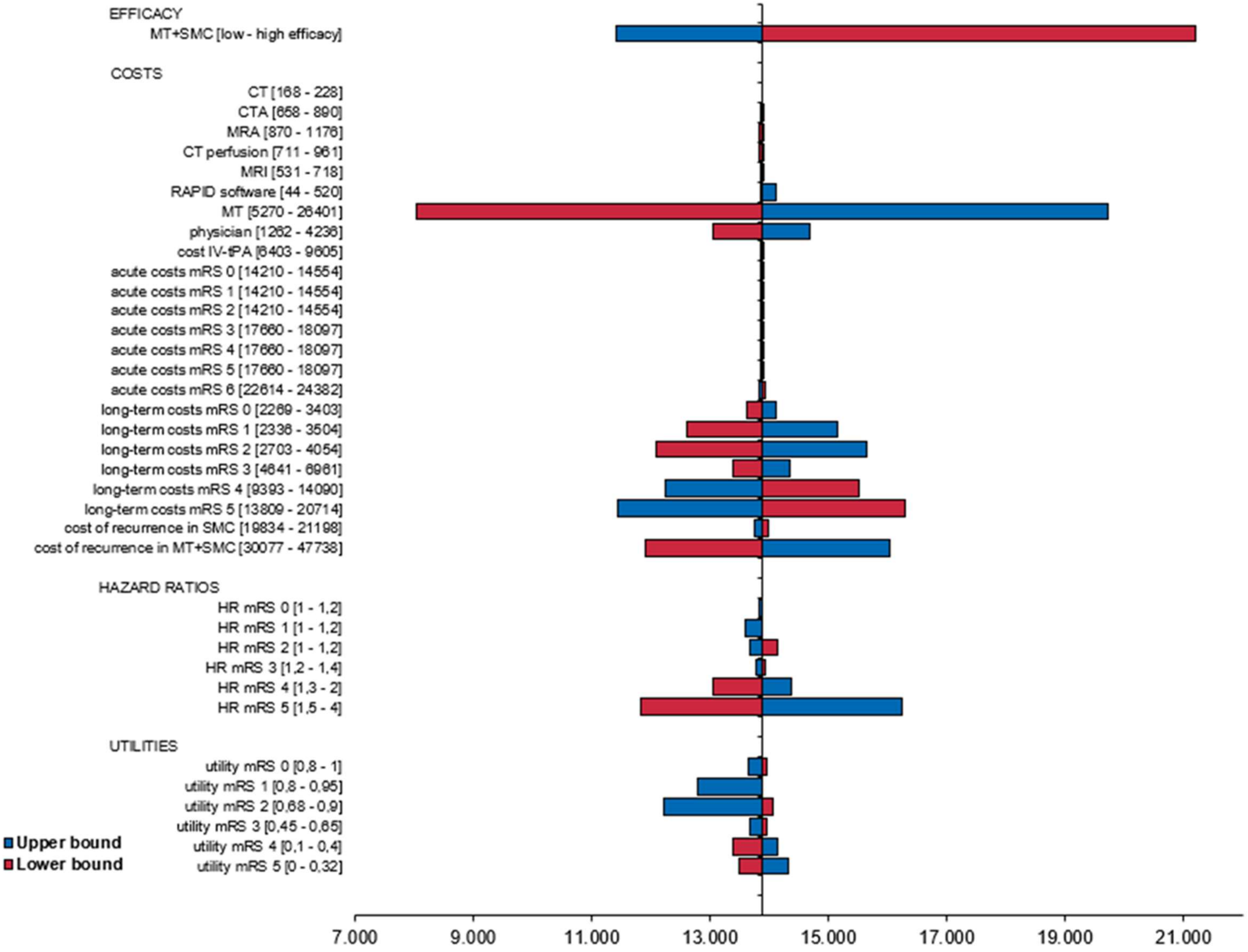

Low and high efficacy of MT+SMC was defined by the following distribution of patients on the mRS score at 3 months:

Low efficacy, by mRS score 0-6 (%): 8.2, 14.2, 16.2, 15, 19.3, 11.2, 16.1

High efficacy, by mRS score 0-6 (%): 11.2, 17.2, 19.2, 15, 17.3, 7.2, 13.2

© 2020 Peultier A-C et al. *JAMA Network Open.*

eFigure 3. PSA Results, Cost-effectiveness Acceptability Curves Showing the Probability of MT With SMC Being Cost-effective at Different Values of WTP for a QALY, by Subgroups

3a

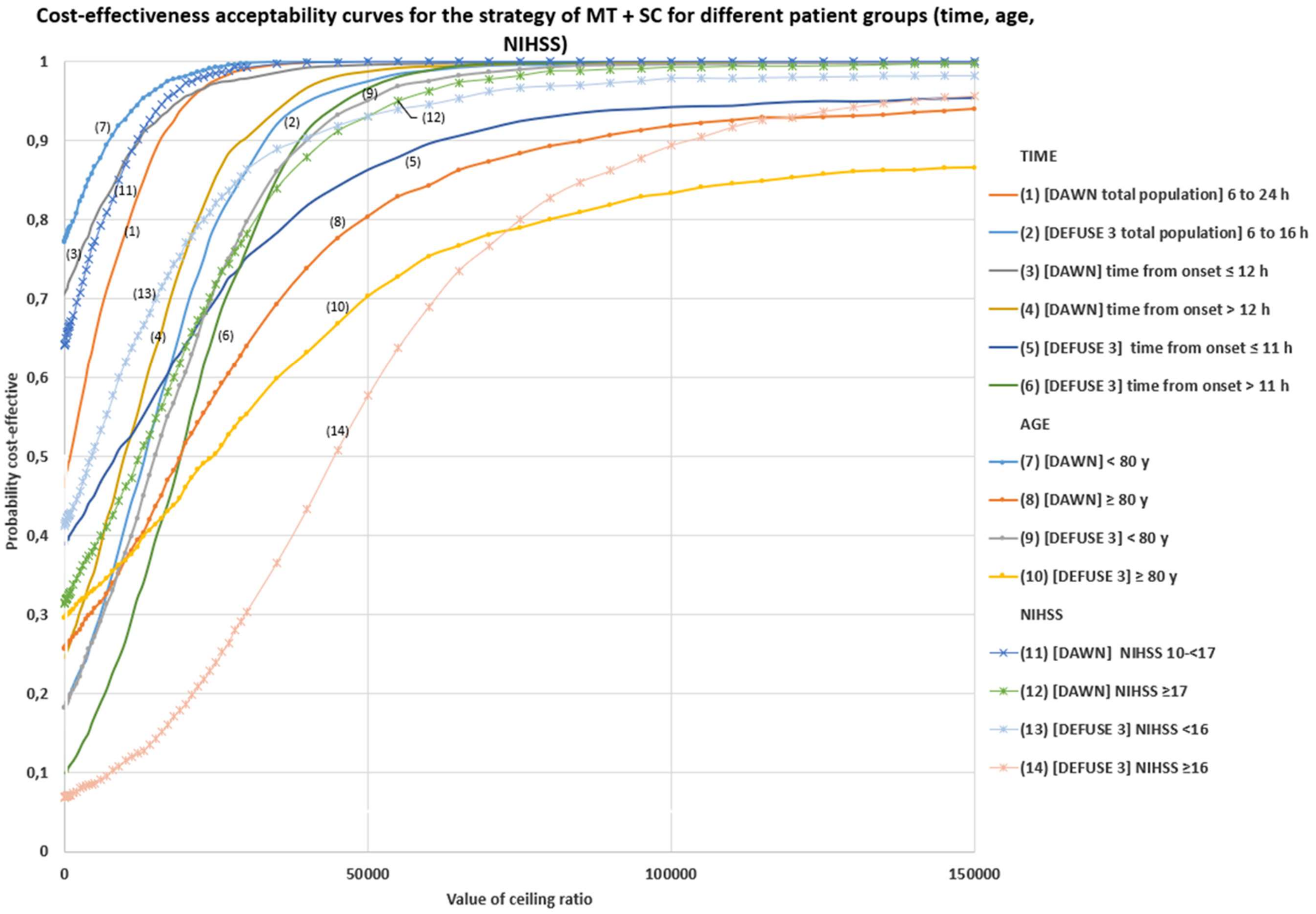

© 2020 Peultier A-C et al. *JAMA Network Open.*

3b

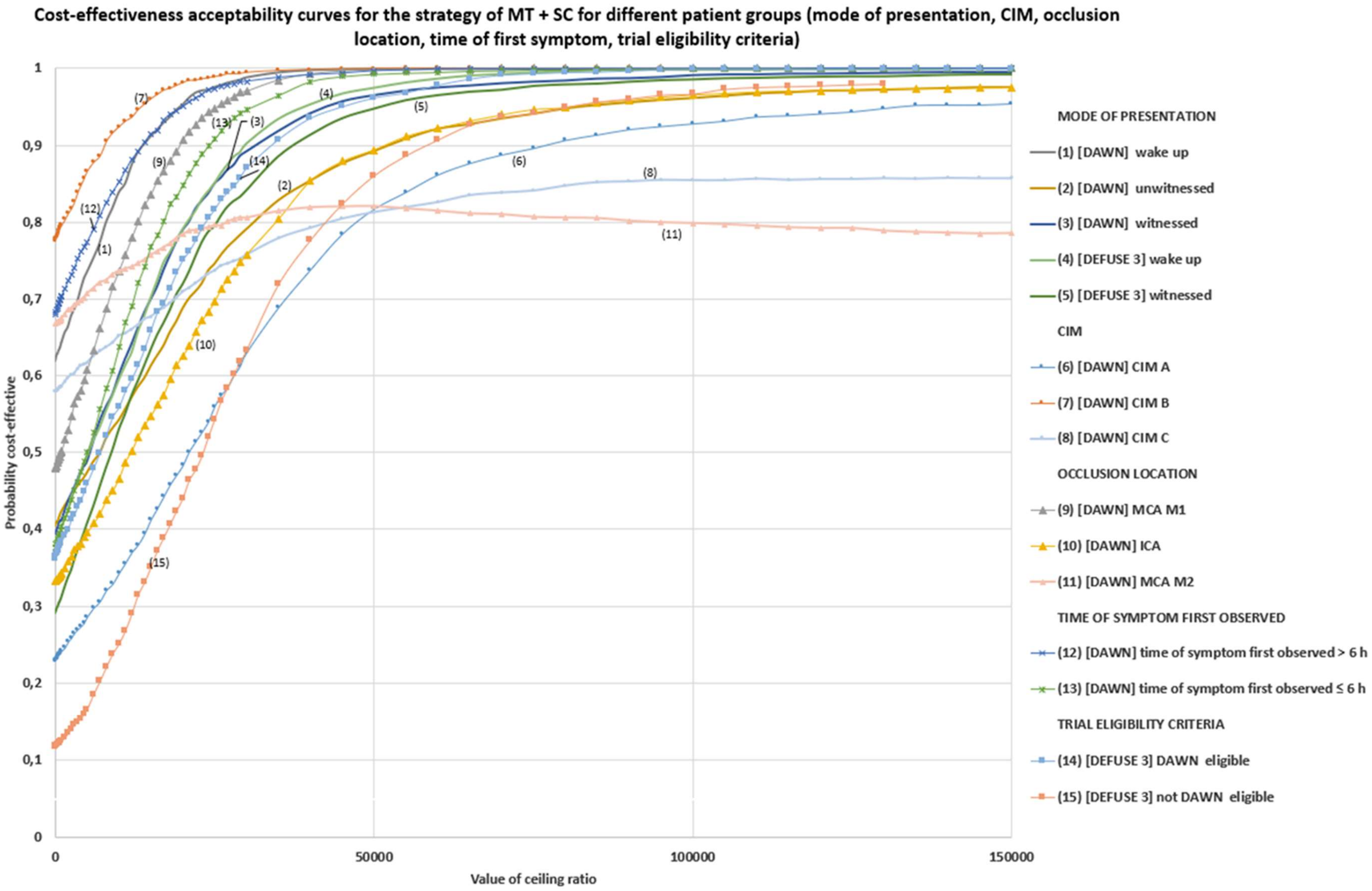

© 2020 Peultier A-C et al. *JAMA Network Open.*

eTable 1. Short-run Model Input Parameters: Distribution of Patients on the mRS Scale at 3 Months After Initial AIS per Subgroup and Strategy per Trial

			DAWN results							
			Distribution at 3 months on the mRS scale							sample size
**Criteria**	**Patient subgroup**	**Strategy**	**mRS 0**	**mRS 1**	**mRS 2**	**mRS 3**	**mRS 4**	**mRS 5**	**mRS 6**	
**Time from stroke onset**	**6 ≤ 24 hours (total population)**									
		SMC	4,1%	5,1%	4,1%	16,1%	34,1%	13,6%	22,6%	99
		MT + SMC	9,1%	22,1%	17,1%	13,1%	13,1%	9,5%	15,8%	107
	**6 ≤ 12 hours**									
		SMC	6,9%	6,9%	5,9%	10,9%	36,9%	12,2%	20,5%	46
		MT + SMC	14%	22%	18%	10%	6%	11,3%	18,8%	50
	**>12 hours**									
		SMC	1,9%	3,9%	1,9%	20,9%	31,9%	14,9%	24,9%	53
		MT + SMC	5%	23%	16%	16%	19%	7,9%	13,1%	57
**Age**	**<80 years**									
		SMC	6%	6%	5%	17%	40%	9,8%	16,3%	70
		MT + SMC	8,9%	25,9%	18,9%	11,9%	14,9%	7,4%	12,4%	82
	**≥ 80 years**									
		SMC	0%	3,8%	0%	13,8%	20,8%	23,1%	38,6%	29
		MT + SMC	12%	12%	8%	16%	8%	16,5%	27,5%	25
**NIHSS**	**10<17**									
		SMC	9,1%	9,1%	6,1%	18,1%	33,1%	9,1%	15,1%	45
		MT + SMC	16,9%	24,9%	28,9%	7,9%	7,9%	5,1%	8,6%	52
	**≥ 17**									
		SMC	2%	0%	2%	15%	35%	17,3%	28,8%	54
		MT + SMC	2,1%	20,1%	5,1%	18,1%	18,1%	13,6%	22,6%	55
**Mode of presentation**	**Wake up**									
		SMC	5%	4%	2%	17%	38%	12,8%	21,3%	47
		MT + SMC	11%	22%	16%	15%	12%	9%	15%	67
	**Unwitnessed**									
		SMC	3%	5%	5%	18%	29%	15%	25%	38
		MT + SMC	7%	24%	10%	7%	17%	13,1%	21,9%	29
	**Witnessed**									
		SMC	7%	7%	7%	7%	36%	13,5%	22,5%	14
		MT + SMC	9%	18%	37%	18%	9%	3,4%	5,6%	11
**Clinical Infarct Mismatch (CIM)**	**CIM Group A**									
		SMC	0%	3,8%	0%	13,8%	20,8%	23,1%	38,6%	29
		MT + SMC	12%	12%	8%	16%	8%	16,5%	27,5%	25
	**CIM Group B**									
		SMC	7%	7%	6%	20%	39%	7,9%	13,1%	61
		MT + SMC	10%	26%	21%	12%	15%	6%	10%	73
	**CIM Group C**									
		SMC	0%	0%	0%	0%	44%	21%	35%	9
		MT + SMC	0%	22,2%	11,2%	11,2%	11,2%	16,7%	27,7%	9

© 2020 Peultier A-C et al. *JAMA Network Open.*

			DAWN results							
			Distribution at 3 months on the mRS scale							sample size
**Criteria**	**Patient subgroup**	**Strategy**	**mRS 0**	**mRS 1**	**mRS 2**	**mRS 3**	**mRS 4**	**mRS 5**	**mRS 6**	
**Occlusion location**	**Internal Carotid Artery (ICA)**									
		SMC	0%	0%	0%	21%	21%	21,8%	36,3%	19
		MT + SMC	4,9%	26,9%	13,9%	13,9%	4,9%	13,4%	22,4%	22
	**Middle Cerebral Artery M1 Segment**									
		SMC	5%	7%	5%	14%	38%	11,6%	19,4%	77
		MT + SMC	10,9%	20,9%	17,9%	12,9%	15,9%	8,1%	13,6%	83
	**Middle Cerebral Artery M2 Segment**									
		SMC	0%	0%	0%	33,3%	33,3%	12,7%	20,9%	3
		MT + SMC	50%	0%	0%	0%	0%	18,8%	31,3%	2
**Time of symptom first observed**	**Symptom First Observed ≤ 6 hours**									
		SMC	2%	6%	5%	20%	32%	13,1%	21,9%	54
		MT + SMC	10%	23%	13%	14%	16%	9%	15%	74
	**Symptom First Observed > 6 hours**									
		SMC	7%	4%	2%	11%	38%	14,3%	23,8%	45
		MT + SMC	9,1%	21,1%	24,1%	12,1%	6,1%	10,3%	17%	33

			DEFUSE 3 results							
			Distribution at 3 months on the mRS scale							sample size
**Criteria**	**Patient subgroup**	**Strategy**	**mRS 0**	**mRS 1**	**mRS 2**	**mRS 3**	**mRS 4**	**mRS 5**	**mRS 6**	
**Time from stroke onset**	**6 ≤16 hours (total population)**									
		SMC	7,9%	3,9%	3,9%	15,9%	26,9%	15,9%	25,9%	90
		MT + SMC	10,1%	16,1%	18,1%	15,1%	18,1%	8,1%	14,1%	92
	**6 ≤11 hours**									
		SMC	11,9%	5,9%	5,9%	15,9%	24,9%	17,9%	17,9%	51
		MT + SMC	12,3%	14,3%	16,3%	12,3%	20,3%	8,3%	16,3%	49
	**>11 hours**									
		SMC	2,9%	2,9%	2,9%	14,9%	27,9%	12,9%	35,9%	39
		MT + SMC	6,9%	18,9%	20,9%	18,9%	15,9%	6,9%	11,9%	43
**Age**	**<80 years**									
		SMC	10,9%	5,9%	4,9%	19,9%	23,9%	14,9%	19,9%	66
		MT + SMC	12,7%	15,7%	22,7%	15,7%	18,7%	5,7%	8,7%	70
	**≥ 80 years**									
		SMC	0%	0%	4%	4%	33%	17%	42%	24
		MT + SMC	0%	17,8%	4,8%	13,8%	17,8%	13,8%	31,8%	22

		DEFUSE 3 results							
		Distribution at 3 months on the mRS scale							sample size
**Criteria**	**Strategy**	**mRS 0**	**mRS 1**	**mRS 2**	**mRS 3**	**mRS 4**	**mRS 5**	**mRS 6**	

© 2020 Peultier A-C et al. *JAMA Network Open.*

**NIHSS**	**<16**								
	SMC	15,9%	8,9%	8,9%	21,9%	17,9%	15,9%	10,9%	45
	MT + SMC	18,9%	22,9%	20,9%	11,9%	11,9%	6,9%	6,9%	43
	**≥16**								
	SMC	0%	0%	0%	8,8%	35,8%	15,8%	39,8%	45
	MT + SMC	2,3%	10,3%	16,3%	18,3%	24,3%	8,3%	20,3%	49
**Mode of presentation**	**Wake up**								
	SMC	5%	0%	2%	17%	26%	19%	31%	42
	MT + SMC	8,3%	16,3%	18,3%	14,3%	16,3%	6,3%	20,3%	49
	**Time known**								
	SMC	8,9%	5,9%	8,9%	10,9%	28,9%	16,9%	19,9%	35
	MT + SMC	16%	19%	23%	10%	13%	13%	6%	31
**Trial eligibility criteria**	**Not DAWN eligible**								
	SMC	8,9%	5,9%	8,9%	8,9%	23,9%	11,9%	31,9%	34
	MT + SMC	11%	19%	25%	6%	17%	8%	14%	36
	**DAWN eligible**								
	SMC	6,9%	3,9%	1,9%	19,9%	28,9%	17,9%	20,9%	56
	MT + SMC	9,1%	14,1%	14,1%	21,1%	20,1%	7,1%	14,1%	56

‡ Clinical infarct mismatch: mismatch between the severity of the clinical deficit and the infarct volume defined according to the following groups:

Group A: age ≥80, NIHSS ≥10, infarct volume <21ml

Group B: age< 80, NIHSS ≥10, infarct volume <31ml

Group C: age< 80, NIHSS ≥20, infarct volume <31-51ml

§ Time of symptom first observed to randomization

ICA: Internal carotid artery

MCA M1: middle cerebral artery M1 segment

NIHSS: National Institutes of Health Stroke Scale

© 2020 Peultier A-C et al. *JAMA Network Open.*

eTable 2. Reported Age of Randomized Patients in the DAWN and DEFUSE 3 Trials and Parameterized Age of Patients in our Model

**Total study population**	**SMC + MT**		**SMC alone**	
	Data from the trial	Data used in our model*	Data from the trial	Data used in our model*
DAWN	69.4 (SD=14.1)	69	70.7 (SD=13.2)	71
DEFUSE 3	70	70	71	71
**Age ≥80 year**	**SMC + MT**		**SMC alone**	
	Data from the trial	Data used in our model (mean age of the patients ≥80)*	Data from the trial	Data used in our model (mean age of the patients ≥80)*
DAWN	23%	86.3 rounded to 86	29%	86.2 rounded to 86
DEFUSE 3	23.9%**	86.6 rounded to 87	26.6%**	86
**Age <80 year**	**SMC + MT**		**SMC alone**	
	Data from the trial	Data used in our model (mean age of the patients <80)*	Data from the trial	Data used in our model (mean age of the patients <80)*
DAWN	77%	65.4 rounded to 66	71%	66.6 rounded to 67
DEFUSE 3	76.1%**	65.7 rounded to 66	73.3%**	66.7 rounded to 67

*estimated assuming a normal distribution around the mean age of the full randomized population (tool: http://davidmlane.com/hyperstat/z_table.html)

** calculated from the data provided in the DAWN and DEFUSE 3 studies

© 2020 Peultier A-C et al. *JAMA Network Open.*

eTable 3. Calculation Methods for the Cost of Software per Ischemic Stroke Patient

	**Mean (range)**	**Comments**	Reference
**Thrombectomy-capable centers**	53	Minimum 15 patients/year	The Joint Commission Website (1)
**Comprehensive stroke centers (CSC)**	194	-	
**Annual stroke patients**	795,000		(2)
**Annual ischaemic stroke patients (87%)**	691,650		
**Annual ischaemic stroke patients in the 6-23-hour onset-to-door window (those likely to receive advanced-imaging and to require the use of RAPID software)**	~10.5%*691,650<br>= 72,623	Of all ischaemic stroke patients:<br>3.0% presented >24 hours 53.0% did not have exact time of onset documented<br><br>Of the 44% remaining, 25.4% were in the 6 to 24-hour onset-to-door window. Assuming linearity, this is 24% in the 6-23-hour window.<br>(1 hour is assumed between arrival at hospital and imaging assessment).<br><br>44%*24%=10.5%<br>(probably an overestimate if there are other contraindications or obstacles to MT for patients within this time window)	(3)
**Annual ischaemic stroke patients in the 6-23-hour window/stroke center**	72,623 / 247<br>= 294 (100 – 400)	Range assumed	
	**Cost (range)**		
**RAPID software**<br>**Annual cost per facility**	$26,250 ($17,500 - 52,000)	depending on configuration (1 scanner or unlimited)	feedback from RAPIDAI (VP sales contact)<br>and from one hospital in the US (for the upper bound)
**RAPID software**<br>**Cost/ischaemic patient (6-24-hour window)/scan**	$89 ($44 – 520)		

© 2020 Peultier A-C et al. *JAMA Network Open.*

eTable 4. Reported Frequencies of Use of Intravenous Thrombolysis in the DAWN and DEFUSE 3 Trials

	**DAWN**	**DEFUSE 3**
**Intervention (MT+SMC)**	5%	11%
**Control (SMC alone)**	13%	9%

© 2020 Peultier A-C et al. *JAMA Network Open.*

eTable 5. PSA Results, Monte Carlo Simulations of Incremental Cost and Incremental QALY per Patient with patient of MT With SMC vs SMC alone

Probabilistic sensitivity analysis per patient group and all subgroups of the DAWN and DEFUSE 3 trials.

**TIME FROM SYMPTOM ONSET**
**when performed within 6 to 24 hours (DAWN) and when performed within 6 to 16 hours (DEFUSE 3) (total population of both studies)**
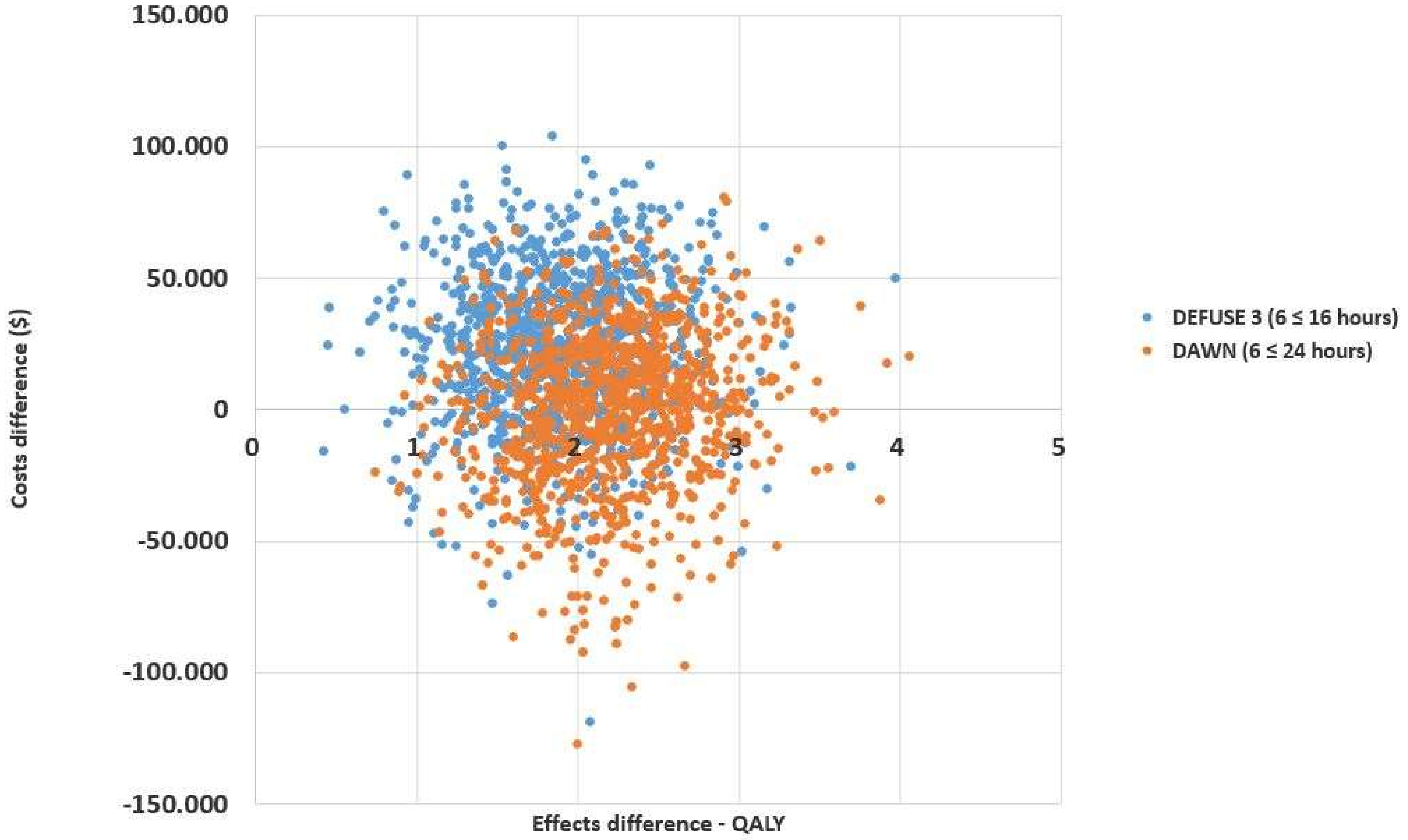

© 2020 Peultier A-C et al. *JAMA Network Open.*

TIME FROM SYMPTOM ONSET	
**when performed within 12 hours compared to beyond 12 hours (DAWN)**	**when performed within 11 hours compared to beyond 11 hours (DEFUSE 3)**

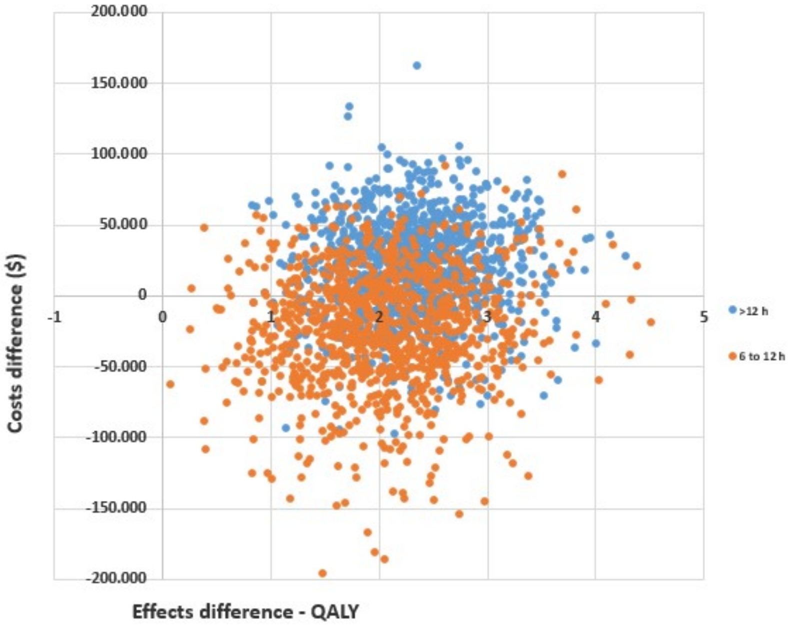

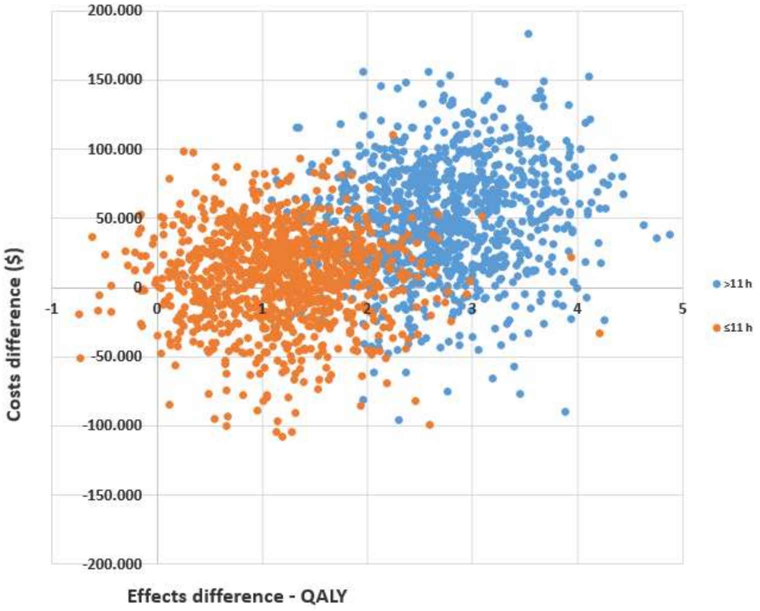

© 2020 Peultier A-C et al. *JAMA Network Open.*

<table>
<tr><th colspan="2">AGE
when performed within or beyond 80 years of age</th></tr>
<tr><th>DAWN</th><th>DEFUSE 3</th></tr>
<tr><td>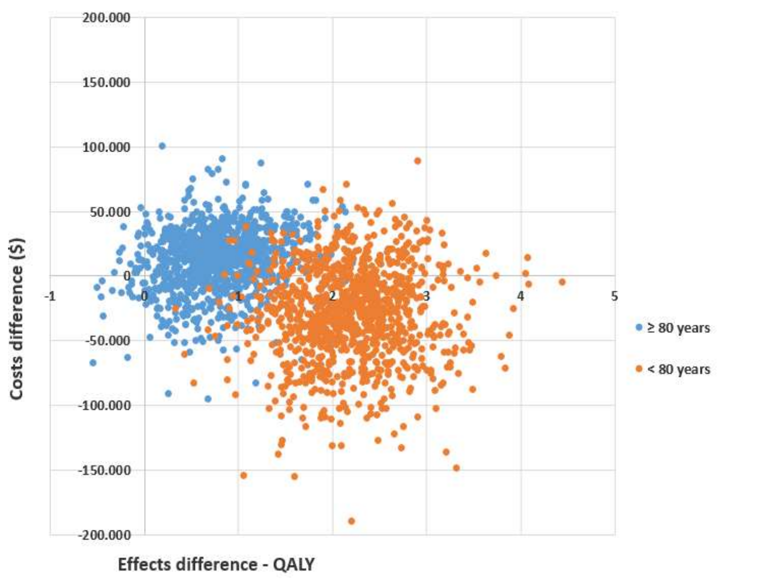
</td><td>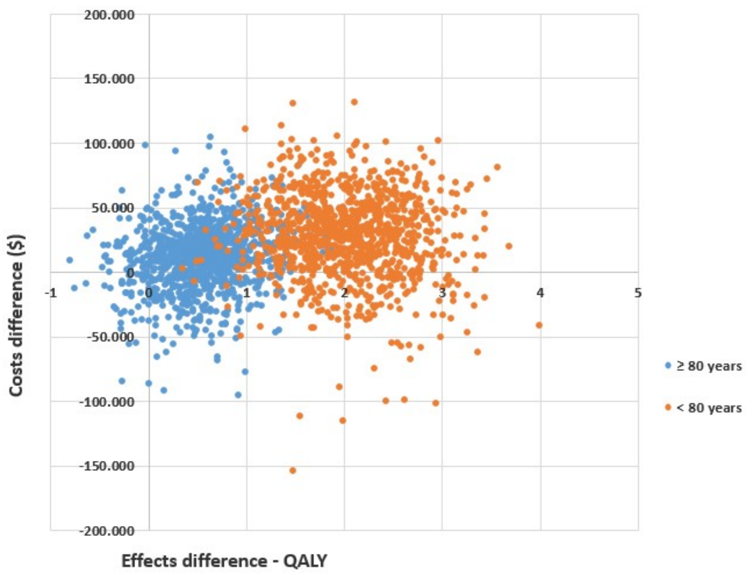
</td></tr>
<tr><th colspan="2">NIHSS</th></tr>
<tr><th>when performed in patients with a baseline NIHSS of 10 to 17 versus beyond (DAWN)</th><th>when performed in patients with a baseline NIHSS of 16 or less versus beyond (DEFUSE 3)</th></tr>
<tr><td>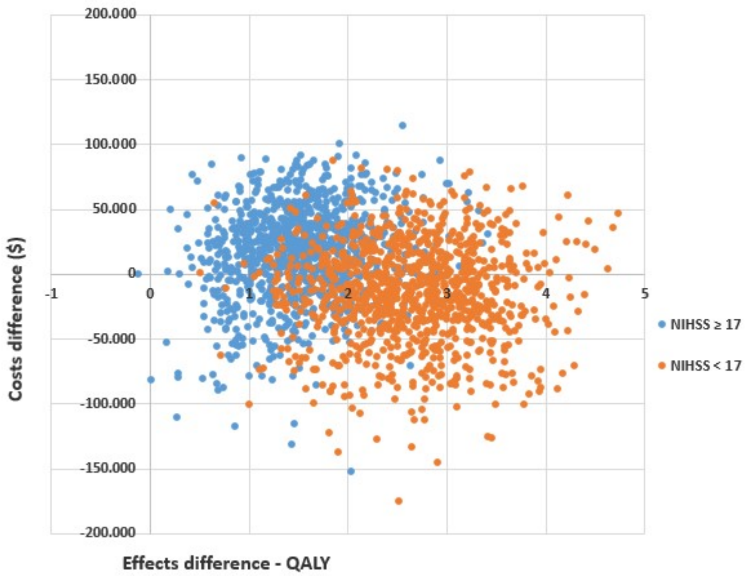
</td><td>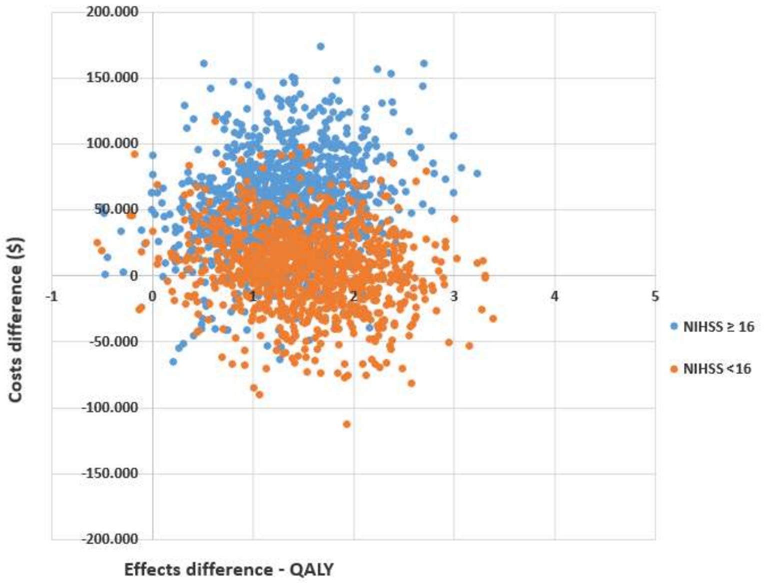
</td></tr>
</table>

© 2020 Peultier A-C et al. *JAMA Network Open.*

MODE OF PRESENTATION

when performed in patients with wake-up stroke versus unwitnessed stroke (DAWN)

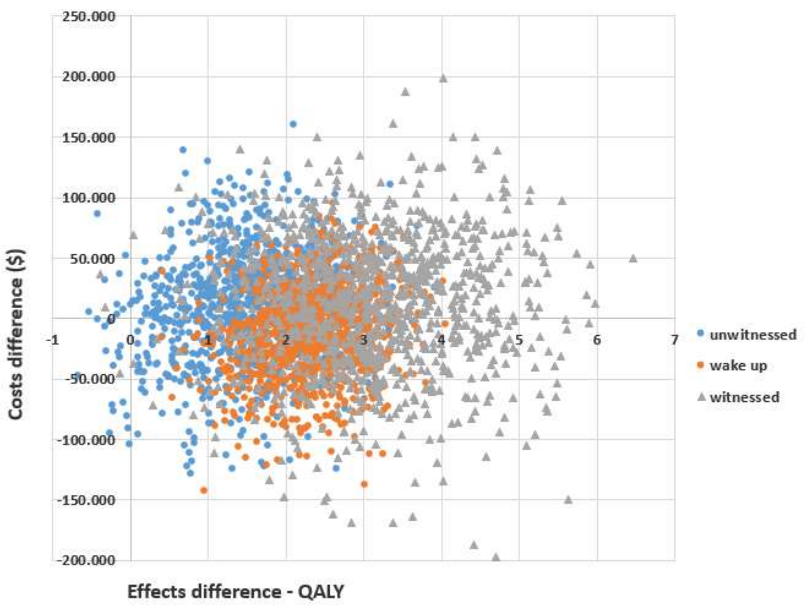

when performed in patients with wake-up stroke versus witnessed stroke (DEFUSE 3)

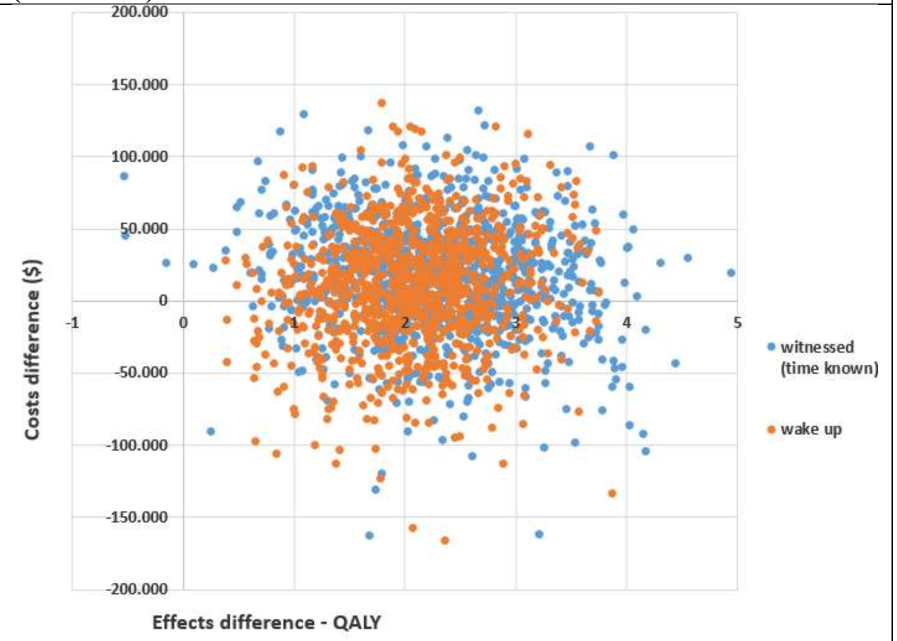

© 2020 Peultier A-C et al. *JAMA Network Open.*

CLINICAL INFARCT MISMATCH (DAWN)
when performed in patients with a specific infarct mismatch versus another one

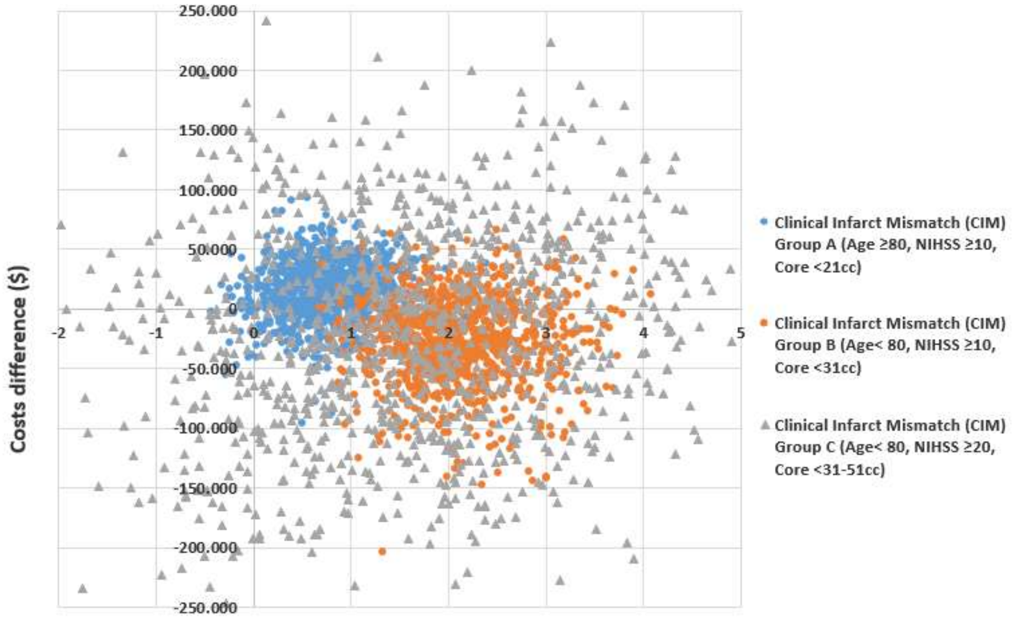

OCCLUSION LOCATION (DAWN)
when performed in patients with MCA M1 occlusion versus ICA

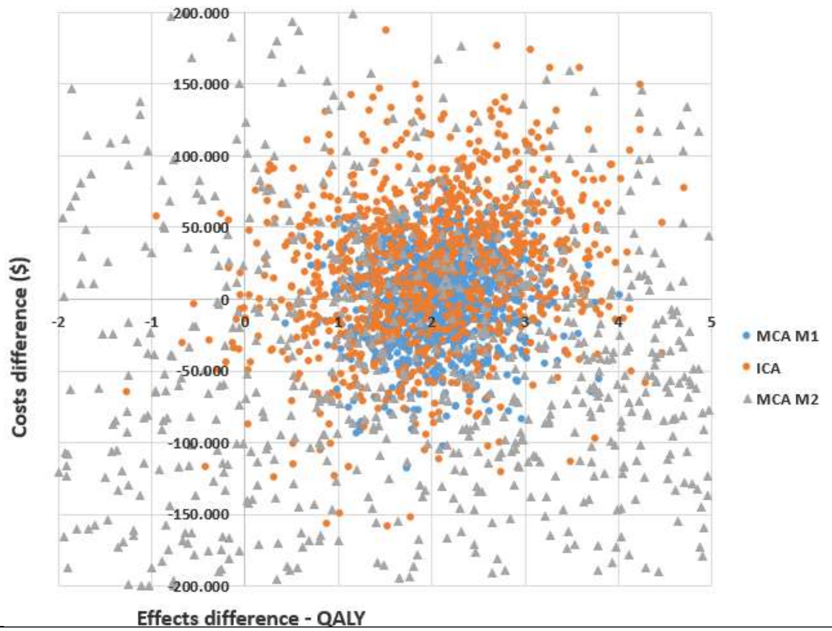

© 2020 Peultier A-C et al. *JAMA Network Open.*

**TIME OF SYMPTOM FIRST OBSERVED (DAWN)**<br>**when performed within or beyond 6 hours of symptom first observed**
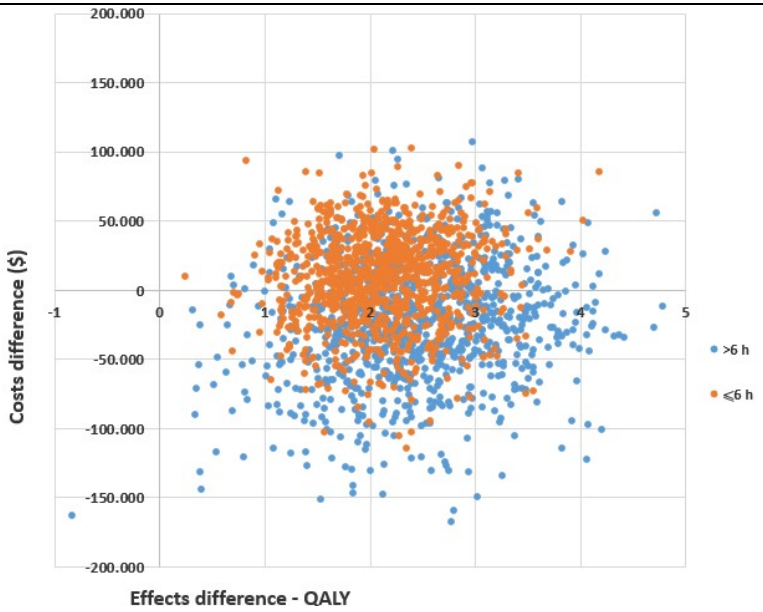
**TRIAL ELIGIBILITY CRITERIA (DEFUSE 3)**<br>**when performed in patients eligible to the DAWN trial versus those non eligible**
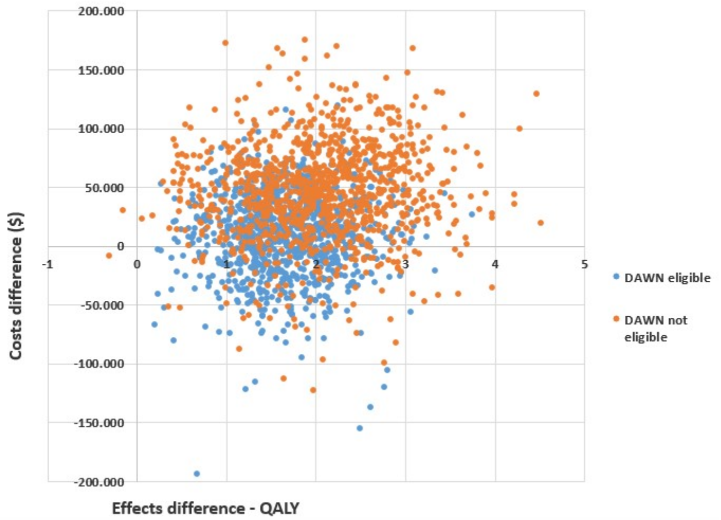

© 2020 Peultier A-C et al. *JAMA Network Open.*

eReferences

1. Joint Commission. Facts about Joint Commission stroke certification. https://www.qualitycheck.org/search/?keyword=connecticut#keyword=connecticut&advancedcertification=Advanced%20Comprehensive%20Stroke%20Center. Accessed April, 01, 2020.

2. Mozaffarian D, Benjamin EJ, Go AS, et al. Executive Summary: Heart Disease and Stroke Statistics—2016 Update. A Report From the American Heart Association. Circulation 2016;133:e38–e360.795,000

3. Tong D, Reeves MJ, Hernandez AF, et al. Times From Symptom Onset to Hospital Arrival in the Get With The Guidelines–Stroke Program 2002 to 2009: Temporal Trends and Implications. Stroke. 2012; 43:1912–1917.

© 2020 Peultier A-C et al. *JAMA Network Open.*
